# Assessment of the FRET-based *Teen* sensor to monitor ERK activation changes preceding morphological defects in a RASopathy zebrafish model and phenotypic rescue by MEK inhibitor

**DOI:** 10.1186/s10020-024-00807-w

**Published:** 2024-04-09

**Authors:** Giulia Fasano, Stefania Petrini, Valeria Bonavolontà, Graziamaria Paradisi, Catia Pedalino, Marco Tartaglia, Antonella Lauri

**Affiliations:** 1 Molecular Genetics and Functional Genomics, Ospedale Pediatrico Bambino Gesù, IRCCS, Rome, 00146 Italy; 2 Microscopy facility, Research laboratories, Ospedale Pediatrico Bambino Gesù, IRCCS, Rome, 00146 Italy; 3 Department for Innovation in Biological Agro-food and Forest systems (DIBAF), University of Tuscia, Viterbo, 01100 Italy

**Keywords:** RASopathies, ERK, Zebrafish embryos, FRET

## Abstract

**Background:**

RASopathies are genetic syndromes affecting development and having variable cancer predisposition. These disorders are clinically related and are caused by germline mutations affecting key players and regulators of the RAS-MAPK signaling pathway generally leading to an upregulated ERK activity. Gain-of-function (GOF) mutations in *PTPN11*, encoding SHP2, a cytosolic protein tyrosine phosphatase positively controlling RAS function, underlie approximately 50% of Noonan syndromes (NS), the most common RASopathy. A different class of these activating mutations occurs as somatic events in childhood leukemias.

**Method:**

Here, we evaluated the application of a FRET-based zebrafish ERK reporter, *Teen*, and used quantitative FRET protocols to monitor non-physiological RASopathy-associated changes in ERK activation. In a multi-level experimental workflow, we tested the suitability of the *Teen* reporter to detect *pan*-embryo ERK activity correlates of morphometric alterations driven by the NS-causing Shp2^D61G^ allele.

**Results:**

Spectral unmixing- and acceptor photobleaching (AB)-FRET analyses captured pathological ERK activity preceding the manifestation of quantifiable body axes defects, a morphological pillar used to test the strength of SHP2 GoF mutations. Last, the work shows that by multi-modal FRET analysis, we can quantitatively trace back the modulation of ERK phosphorylation obtained by low-dose MEK inhibitor treatment to early development, before the onset of morphological defects.

**Conclusion:**

This work proves the usefulness of FRET imaging protocols on both live and fixed *Teen* ERK reporter fish to readily monitor and quantify pharmacologically- and genetically-induced ERK activity modulations in early embryos, representing a useful tool in pre-clinical applications targeting RAS-MAPK signaling.

**Supplementary Information:**

The online version contains supplementary material available at 10.1186/s10020-024-00807-w.

## Introduction

Extracellular signal-regulated kinases (ERK) 1 and 2 are the last tiers of the mitogen-activated protein kinase (MAPK) signaling cascade, a major evolutionary conserved effector pathway of RAS proteins. The cascade translates a wide array of morphogens’ inputs, thereby controlling several cellular processes, such as proliferation, survival, migration, and differentiation, during critical developmental windows (Gotoh et al. 1991; Krens et al. 2006, 2008a; Shaul and Seger 2007). Upon growth factor stimulation and autophosphorylation of activated cell surface receptor tyrosine kinases, RAS proteins are activated by guanine nucleotide exchange factors that are recruited to the membrane. RAS activation also requires proper function of SHP2, a cytoplasmic protein tyrosine phosphatase acting as a positive regulator of the pathway (Tartaglia et al. 2004b; Tajan et al. 2015) by dephosphorylating regulatory tyrosine residues representing docking sites for proteins negatively controlling RAS function (Dance et al. 2008).

Somatic mutations in several genes encoding the three members of the RAS subfamily, their effectors, and several positive and negative modulators of RAS function represent the most common event driving oncogenesis (COSMIC database, https://cancer.sanger.ac.uk/cosmic). Mounting genetic evidence of the last two decades showed that germline mutations in these genes also underlie a family of developmental disorders collectively called “RASopathies” (Tartaglia and Gelb, 2010; Rauen 2013; Tartaglia et al. 2022). Despite the different genes and mutations involved, these disorders are clinically related, the majority sharing upregulation of signal flow through the RAS-MAPK pathway during development. Major shared features include a distinctive craniofacial gestalt, post-natal short stature, developmental delay, variable cognitive deficits, congenital heart defects and hypertrophic cardiomyopathy (HCM), skeletal defects and ectodermal anomalies (Tidyman and Rauen 2009; Tartaglia et al. 2011; Rauen 2013; Jindal et al. 2017), testifying the importance of a correct RAS-MAPK signaling for proper development.

Germline gain-of-function (GoF) mutations in *PTPN11*, encoding SHP2, account for 50% of Noonan syndrome (NS) (Tartaglia et al. 2001, 2002), the most common and clinically variable among RASopathies (Tartaglia et al. 2011). Mutations are almost always missense changes and perturb SHP2’s function through distinct mechanisms, with the majority enhancing SHP2’s function by impairing the switch between the active and inactive states, favoring a shift in the equilibrium toward the former (Tartaglia et al. 2004b). Mutations in *PTPN11* are more prevalent among patients showing pulmonic stenosis and short stature, while are rarely associated with hypertrophic cardiomyopathy, and severe cognitive impairment, which are more common among patients carrying mutations in other genes acting downstream in the pathway (e.g., RAF1, RIT1, MRAS, and LZTR1) (Tartaglia et al. 2002, 2022; Pierpont 2015). Nevertheless, patients with *PTPN11* mutations can show learning and memory deficit (Pierpont 2015), which is likely linked to the role of SHP2 during neurodevelopment, particularly in corticogenesis (Yamamoto et al. 2005). Consistently, functional data in mice demonstrate that the NS-associated D61G amino acid substitution causing GoF of SHP2 and enhanced RAS-MAPK signaling impairs the balance between gliogenesis and neurogenesis in the developing cortex (Gauthier et al. 2007; Ehrman et al. 2014).

Moreover, children with NS are predisposed to a spectrum of hematologic abnormalities and malignancies, including juvenile myelomonocytic leukemia (JMML) (Niemeyer 2014). A distinct class of mutations in this gene are acquired as somatic events and occur in approximately one-third of children with isolated JMMLas well as variable proportions of other childhood myeloid and lymphoid malignancies (Tartaglia et al. 2003, 2004a). These mutations alter residues located at the interface between the N-SH2 and PTP domains but are more activating compared to germline variants and are not compatible with embryonic/fetal development (Keilhack et al. 2005; Tartaglia et al. 2006).

During the last two decades, > 20 genes have been causally linked to RASopathies (Tartaglia et al. 2022). We now know that in these disorders, increased RAS-MAPK signaling can result from the upregulated activity of various GTPases of the RAS family, increased function of signal transducers positively controlling RAS activity or favoring RAS interactions with RAF kinases, functional upregulation of the three tiers of the MAPK cascade, or inefficient signaling switch-off operating at different levels. Notably, most RASopathy-causing mutations show functional convergence operating at the level of RAS and RAF proteins (Tartaglia et al. 2022).

Poor therapeutic options are currently available for patients affected with RASopathies. To this goal, an incremental understanding of the molecular mechanisms underlying these disorders is a prerequisite to approaching targeted therapies aimed at ameliorating or treating progressive complications of these disorders (Gelb et al. 2022; Hebron et al. 2022).

Experiments in mice (Chen et al. 2010; Hernández-Porras et al. 2014; Inoue et al. 2014) and fish (Runtuwene et al. 2011; Bonetti et al. 2014a; Solman et al. 2022; Anastasaki et al. 2012) suggest the efficacy of inhibiting the RAS/MAPK signal via MEK inhibitors (MEKi) administration in preventing RASopathy-associated developmental defects. From here, MEKi have been proposed as a possible therapeutic intervention in clinical practice (Andelfinger et al. 2019), and were successfully applied to treat RASopathy-associated HCM and lymphedema (Gelb et al. 2022). Similarly, animal model systems have been used to validate also targeted therapeutic approaches (Lee et al. 2014; Bobone et al. 2021; Das et al. 2021). However, further pre-clinical studies to clarify the current uncertainty about treatment windows and specific phenotypic rescue correlated to ERK activity modulation in developing tissues are necessary for the effective use of the available MEKi in clinical practice.

The use of zebrafish as a vertebrate model offers the possibility of setting up parallel morphological and molecular pre-clinical readouts in vivo. A number of assays can be performed in embryos for carrying out a rapid functional classification of variants of unknown significance (VoUS) and for a detailed study of mutations’ impact on embryogenesis. Zebrafish can be also an informative experimental model system to validate potential therapeutic solutions in terms of dosage and effective treatment windows (Patton et al. 2021).

Zebrafish represents a solid model to investigate the differential impact of both GoF and loss-of-function (LoF) RASopathies-causing mutations (Jopling et al. 2007; Runtuwene et al. 2011; Bonetti et al. 2014a, b; Jindal et al. 2017; Nakagama et al. 2020; Motta et al. 2021). Clear disease features are recapitulated in fish. Morphological parameters can be indeed conveniently scored in young fish mutants to test the impact of the variants and the mechanism of action underlying variable clinical severity. Convergence extension impairment impacting early axes morphogenesis and elongation are often an early disease hallmark scored in RASopathies models in both fish and insects (“oval embryo” assay and measurement of embryo elongation at later stages), consistently with the activity of growth factor-stimulated RAS-MAPK signaling during gastrulation (Delfini et al. 2005; Jopling et al. 2007; Gervaise and Arur 2016; Jindal et al. 2017; Patel et al. 2019; Hayashi and Ogura 2020; Anastasaki et al. 2012) (Supplementary Table 1).

Spatio-temporal ERK phosphorylation dynamics driven by RASopathies-associated variants and modulated by drug treatments in developing embryos can now be studied live thanks to various FRET-based ERK activity sensors developed and tested in vivo (Kamioka et al. 2012; Lauri et al. 2021). In a FRET-based extracellular-regulated kinase reporter (EKAR) type, active ERK (pERK) phosphorylates a substrate within the sensor, which is then translated in a conformational change of the sensor bringing together a donor (D)-acceptor (A) pair (such as CFP and YFP).

When the donor is excited, a FRET phenomenon can then occur, proportional to the distance between donor and acceptor. Donor emission can be indeed absorbed by an acceptor ideally with a high extinction coefficient (such as YFP or YPet), if the donor and acceptor get in close proximity (typically 1–10 nm) and if the emission of the donor overlaps significantly with the absorption spectrum of the acceptor (as in case of CFP-YFP pair). In EKAR sensor, a FRET signal can be then registered in vivo within tissues and quantified, which is proportional to the level of pERK in the cells, a proxy for activated RAS/MAPK-ERK signaling (Komatsu et al. 2011).

Different methods to acquire and quantify FRET signals (Miyawaki 2011; Bajar et al. 2016), that can be applied to EKAR-based sensors, have been developed and proposed. For sensors using classical CFP and YFP pairs and their variants, showing inevitable emission overlap, multispectral acquisitions followed by spectral unmixing modality are the most reliable methods to obtain FRET data. Briefly, upon donor excitation, a lambda (λ) stack acquisition is performed, collecting emission spectra along small wavelength bands (normally ranging from 5 to 12 nm). The collected spectra can be then assessed upon inspection along the wavelength axis to assign the best emission window for the two fluorophores, operating therefore a more accurate “unmixing” of the donor and acceptor emission (Dickinson et al. 2001; Zimmermann et al. 2002; Ecker et al. 2004; Gu et al. 2004).This method, relatively fast for simple x,y images, is not invasive, nor disruptive and is thus well suitable to acquire live specimens.

Another commonly used approach is based on Acceptor photobleaching (AB)-FRET, in which the acceptor is bleached in a given region of interest, resulting in a de-quenching of the donor (increased emission), that is dependent on the energy transfer ability and therefore the distance between donor and acceptor (Zeug et al. 2012). FRET efficiency (E), measuring the proportion of energy that the donor transfers to the acceptor can be directly calculated in modern confocal microscopes with dedicated AB-FRET modules. From here the relative distance between donor and acceptor can be retrieved mathematically (Patterson et al. 2000; Hennigan et al. 2009; Vogel et al. 2014; Algar et al. 2019). Compared to spectral imaging, AB-FRET induces more phototoxicity and is therefore not compatible with live acquisitions. Nevertheless, it is relatively fast as it is usually performed only in *x, y* and it provides direct E measures that can be compared between experimental groups without the need for post-imaging analysis.

While quantitative determination of RAS-MAPK signaling during mice embryogenesis is challenging, FRET-based sensors in vivo in zebrafish, optically transparent and harboring a fast embryonic development, such as the recently developed EKAR-type *Teen* biosensor (Tg[*ef1a:ERK biosensor-nes*] *Teen*) (Wong et al. 2019), are very promising. Poor performance when it comes to the dynamic range, i.e. the ability to register subtle but significant changes, is known for FRET sensors using CFP and YFP-based fluorescent pair (Lam et al. 2012) such as EKAR. *Teen* sensor is built with an optimized backbone known as “EKAREV” that increases the sensitivity of the sensor as demonstrated in cells (Komatsu et al. 2011). Using *Teen* for the first time in vivo in combination with spectral unmixing (Sari et al. 2018; Wong et al. 2019) demonstrated high and dynamic ERK activity in known domains of the developing zebrafish embryos can be captured, enabling continuous readouts and spatio-temporal mapping of ERK activity in live embryos. The registered signal, despite showing little changes, recapitulated known dynamics during gastrulation and segmentation stages, as well as signal changes obtained upon pharmacological modulation, *i.e. *MEKi (van Boxtel et al. 2018). A complementary morpho-molecular approach with spatio-temporal resolution at the *pan*-embryo level is expected to contribute to faster and precise testing of mutants’ strength– and thereby disease sub-type classification– as well as to cogent verification of drug efficacy in vivo. Indeed, a proof of principle approach was obtained by ERK actuators and shown by Patel et al. 2019 (Patel et al. 2019), exploring early *real-time see-through* examination of morphological changes and co-occurring ERK activity fluctuations in early insect and fish embryos. Here, by using complementary quantitative FRET imaging protocols both in live embryos (by “spectral unmixing”) and on fixed specimens (by AB-FRET), we tested the potential of *Teen* biosensor to capture as early as possible spatially-restricted pathogenic ERK activity events (molecular level) correlating with the onset of known axes defects (morphological level) in whole developing fish mutants expressing the NS-causing Asp61Gly (D61G) substitution in Shp2. We examined the effectiveness of low- and high-dose treatment with the MEKi PD0325901, concerning both the morphological and ERK activity rescue. We tested the usefulness of two quantitative FRET protocols, validated in early embryos by standard immunofluorescence against pERK, to assess early drug effect on ERK activation.

## Materials and methods

### Zebrafish husbandry

Wild-type zebrafish (NHGRI) (LaFave et al. 2014) were obtained from EZRC (European Zebrafish Resource Center), and *Tg[ef1α:ERK biosensor-nes] (Teen)* (Wong et al. 2019) were obtained from the National BioResource Project of Japan for Zebrafish (RIKEN, Japan) (Urasaki et al. 2006; Okamoto and Ishioka 2010). Fish were cultured following standard protocols. Briefly, embryos were kept in E3 embryo medium (5 mM NaCl, 0.17 mM KCl, 0.33 mM CaCl_2_ and MgSO_4_). Zebrafish lines were housed in a water-circulating system (Tecniplast©) under controlled conditions (light/dark 14:10, 28 °C, 350–400 µS, pH 6.8–7.2) and fed daily with dry and live food (freshly hatched nauplii of *Artemia salina*). All animal experiments were performed according to standard breeding and ARRIVE guidelines (https://arriveguidelines.org) with the approval of the Italian Ministry of Health (DGSA -Direzione generale della sanità animale e dei farmaci veterinari, code: 23/2019-PR).

### Embryos treatment with SHP099

A pool of *Tg[ef1α:ERK biosensor-nes] (Teen)* zebrafish embryos were collected at 4 hpf and treated until 24 hpf with SHP099 inhibitor (MedChem Express, HY-100,388, 10–15 µM) by bath immersion in a volume of 200 µl per well in a 24 well plate (circa 20 embryos per well). Embryos were kept in the incubator at 28 °C during the time of treatment. All experimental conditions received the same concentrations of DMSO (vehicle control, 0.01%). At 24 hpf control and SHP099-treated embryos destined for western blot analysis were collected in dry ice and kept at -80 °C, embryos destined to AB-FRET (see below) were fixed in 4%PFA in PBS 1x for 1 h at room temperature (RT) and stored in PBS 1X at 4 °C. Embryos destined to live spectral imaging were immediately mounted and imaged as described below.

### Embryos treatment with recombinant EGF

*Tg[ef1α:ERK biosensor-nes] (Teen)* zebrafish embryos at 1 dpf (days post-fertilization) were collected, individually dechorionated and treated with acute exposure to EGF at 1 mg/ml (Gibco, 400-25-1MG, dissolved in H_2_0) by bath immersion in a total volume of 200 µl/per well in a 24 well plate (circa 20 embryos per well). For the western blot experiment, fish at 20 hpf and 24 hpf were assessed. For standard FRET analysis (Jares-Erijman and Jovin 2003) using “Calcium Calculator” module of Leica Sp8 early embryos were used (10 ss, circa 15 hpf) and were immediately mounted and imaged as described below.

### Embryos injection with recombinant EGF

For brain EGF delivery in vivo, 24 hpf embryos loosely mounted in 1.5% low-melting agarose (Sigma-Aldrich, A9414) dissolved in embryo medium (E3) were microinjected with 1–2 nl of a solution containing EGF at 1 mg/ml directly into the brain ventricle using custom-pooled capillaries as previously described (Venditti et al. 2022) and FemtoJet 4x microinjection system (Eppendorf). Live FRET imaging using spectral unmixing was performed before and after injection as described below.

### Shp2 mRNA injection in zebrafish embryos and treatment with the MEK inhibitor PD0325901

The full coding sequence of the zebrafish *shp2*^WT^ and mutant *(shp2*^D61G^) from the pCS2+_eGFP-2a-Shp2a (Bonetti et al. 2014a) were subcloned without GFP tag into pCSDest vector using Gateway cloning (ThermoFisher, 11,789,020, 11,791,020). Plasmid linearization was performed with KpnI-HF enzyme (New England Biolabs, #R3142) and capped *shp2* mRNAs was transcribed using mMACHINE™ SP6 Transcription Kit Poly A Tailing Kit (ThermoFisher, AM1340, AM1350) following manufacturer’s instructions. 60 pg was capped mRNA was injected in one-cell stage *Tg[ef1α:ERK biosensor-nes] (Teen)* zebrafish embryos using FemtoJet 4x microinjection system (Eppendorf). Injected embryos were cultured under standard conditions at 28 °C. For FRET experiments, *Teen* fish were screened for GFP fluorescence at circa 4 hpf. For the treatment with PD0325901 MEK inhibitor, injected embryos were randomly divided in three subgroups of circa 20 embryos at 4 hpf and treated with low (0.25 µM) and high (1 µM) doses. Embryos were raised until circa 5 hpf (for FRET experiments) and 11 hpf (for FRET experiments, body axis analyses and western blots) or until 55 hpf (for body elongation measurements). All experimental conditions received the same concentrations of DMSO (vehicle control, 0.01%). Control embryos (not injected) shown in Supplementary Fig. 7 were treated with 1 µM PD from at 7 hpf.

### Oval embryo test and axes ratio measurements at 11 hpf 

At the end of gastrulation (11 hpf), live Shp2-overexpressing zebrafish embryos were imaged using Leica M205 microscope (Leica Microsystems) with a 2x magnification objective to evaluate the occurrence of oval shape (aberration of major vs. minor axis). Embryo axes ratio (major-to-minor) were measured from the acquired images using Fiji software (Schindelin et al. 2012) employing the “straight line” tool. Following (Motta et al. 2021), the ratio between measured length of the major axis vs. the minor axis was calculated and reported.

### Body elongation assessment in hatched embryos

Hatched embryos (55 hpf) were fixed in 4% PFA/PBS 1x. Specimens were laterally sided in single wells containing 1% PBS and imaged using Leica M205 microscope (Leica Microsystems) with a 2x magnification to assess overall body elongation. Body length was measured from the acquired images using Fiji software and employing the “straight line” tool. Raw body length data were reported.

### Fluorescence resonance energy transfer (FRET) by “acceptor photobleaching” (AB) modality

All the *Tg[ef1α:ERK biosensor-nes] (Teen)* positive zebrafish embryos destined to AB-FRET were previously fixed in 4% PFA/PBS at RT for 1 h and stored in PBS 1X at 4 °C. Fixed specimens were mounted laterally in 2% low-melting agarose (Sigma-Aldrich, A9414) dissolved in PBS 1X. FRET assay experiments were carried out using the AB-FRET module of the LAS X software of the Leica TCS-SP8X confocal microscope (Leica Microsystems) equipped of an Argon laser with 458-476-488-496-514-nm lines (50% of laser power), using a Fluotar Visir 25x/0.95 numerical aperture (NA) water immersion objective, with a 1024 × 1024 image format, an optical zoom of 1.5x, 400 Hz scan speed, and a 16-bit image resolution. The AB-FRET wizard of LAS X software was used for the evaluation of FRET efficiencies. First, 2 pre-bleaching x,y scans were performed in sequential mode using the 458 nm excitation line to acquire the donor CFP signal (465–500 nm capture range of the emitted light, providing pre AB-FRET values) and the 514 nm excitation line to acquire the acceptor YPet signal (520–555 nm capture range). Then specific regions of interest (ROIs) were selected using the ROI tool of the software for the next bleaching step. After 50 bleaching iterations of the acceptor fluorophore using the 514 nm laser line (100% intensity to ensure complete bleaching) in each selected ROI, an imaging *x,y* scan of the donor and acceptor emission (providing post AB-FRET) was performed. For acquisitions at 24 hpf all the ROIs were drawn with similar sizes across samples. For acquisition in early embryos ROI were drawn to assess the margin of the animal pole.

### In vivo spectral FRET imaging

Live embryos were mounted in 1.5% low melting agarose (Sigma-Aldrich, A9414) dissolved in embryo medium (E3) and spectral FRET imaging was performed on a TCS-SP8X confocal microscope (Leica Microsystems) equipped with a stage incubator (OkoLab, Italy) allowing to maintain a stable temperature in the imaging chamber (28 °C) and constant humidity during live cell imaging. Live spectral imaging was performed. Time lapses were acquired with a 30 min (Supplementary video 1) or 13 min time interval. For multi-spectral acquisitions with 30 min time interval, these parameters were used: *xyzλt *scanning mode with the 458 nm excitation laser line (50% of the Argon laser power), operating in the 462–572 nm nm of the spectral range with 5 nm detection bandwidth, 5 nm of λ detection step size and 22 steps. For acquisitions with 13 min time interval, λ detection step size was increased from 5 to 7 nm in order to reduce the number of detection steps (from 22 to 16) while keeping the same emission detection range from 462 to 572 nm.A PLAPO 10x/0.40 NA dry objective was used, images were acquired with 512 × 512 image format and 8 bit image resolution (for gastrulae) or 1024 × 1024 and 16 bit image resolution (for 24 hpf samples), scan speed of 400 Hz, *z*-step size of 8 mm. At the end of the imaging session, CFP and Ypet emission signals were separated and the cross-talk between channels was subtracted using the “Spectral dye separation” tool of the LAS X software, by assigning the following settings for net emission spectra separation: 465–500 nm (donor, CFP) and 525–570 nm (acceptor, YPet). After the CFP and YPet channels’ separation, FRET ratio was calculated using build-in software in LAS X.

### Measurements of in vivo FRET changes in EGF bath-treated 10 ss embryos

Live FRET changes induced by bath stimulation with recombinant EGF (1 mg/ml) were assessed in embryos mounted in 1.5% low-melting agarose dissolved in E3 medium. Embryos are imaged using TCS-SP8X confocal microscope (Leica Microsystems) equipped of a stage incubator (OkoLab, Italy) under controlled conditions (temperature of 28 °C and a humidified atmosphere), using a simple time lapse mode (*x,y,z,t*) scanning mode in the “Calcium calculator” wizard with 1024 × 1024 image format at 400 Hz, a 5 μm *z*-step size and with a time interval of 1 min. The 458 nm laser line (50% of the Argon laser power) was used to excite the CFP donor, and the detection of the two wavelength ranges, one for CFP (465–500 nm) and one for YPet (525–570 nm) emission spectra, respectively.

### FRET data analysis and image rendering

FRET data of zebrafish live and fixed samples were processed using the Leica LASX v. 3.5 and Fiji. Intensity FRET signal from the multi-spectral imaging on live samples and FRET efficiency data from the AB experiments on fixed specimens were obtained using the spectral dye separation and the AB-FRET modules, respectively, implemented within Leica LAS X v.3.5. To analyze changes in FRET signal from the spectral unmixing data, analysis of selected ROI was conducted from sum-intensity *z*-projections of ratiometric images (FRET, YPet, Channel 2) / donor, CFP, Channel 1) from the entire embryos in 8 or 16-bit format.

For AB-FRET, the mean values of FRET efficiency within the bleached ROI were obtained by the following formula: *E= (DΩ-DA)/ DΩ*, where DA is the donor-intensity before bleaching, and DΩ is the donor-intensity after bleaching. Donor-Acceptor (D-A) fluorophore distance (calculated as RDA value) was obtained using the following formula:

$${R_{DA}} = \root 6 \of {{{\bar R_0^6} \over {\left\langle E \right\rangle }} - \bar R_0^6} = {\bar R_0} \cdot \root 6 \of {{1 \over {\left\langle E \right\rangle }} - 1} $$

To obtain RDA values, the Foster distance (R_0_) for YFP-CFP (A-D) FRET pair was assumed to be 4.7 nm as previously reported (Patterson et al. 2000; Hennigan et al. 2009; Vogel et al. 2014).

For image rendering, confocal sum- or max-intensity *z*-projections were generated for both the FRET (YPet, channel 1) and donor (CFP,channel 2) using Fiji dedicated plugins. “Smart” LUT was used to show FRET/CFP ratiometric image and brightness, contrast, smoothing and noise correction parameters were adjusted equally within the image and among samples belonging to the same experiment.

### pERK levels detection via immunoblots on embryos protein extracts

Embryos were collected on dry ice and stored at − 80 °C. Total protein lysates were obtained by sonication in cold RIPA lysis buffer (150 mM NaCl, 50 mM TrisHCl pH 8.1% Triton x-100, 0.5% Sodium deoxycholate, 0.1% SDS, ddH_2_O) containing inhibitor cocktail 2 and 3 protein phosphatases (Sigma-Aldrich, P5726, P0044) and Roche cOmplete Mini EDTA-free protease tablets (Roche, 1,183,617,000). Equal amounts of protein extracts ($\sim$ 30 µg) were separated on a 10% Sodium dodecyl sulfate (SDS)-polyacrylamide gel. The total protein concentration was determined by the Bradford assay (Bio-Rad, #5,000,205) using Infinite M Plex (Tecan). After electrophoresis, proteins were transferred to Trans-Blot Turbo Mini 0.2 μm nitrocelluloseTransfer Packs (Biorad, #1,704,158) using Trans-Blot Turbo Transfer System (Biorad). Blots were blocked with 5% non-fat milk powder (diluted in TBST 1X, Santa Cruz Biotechnology, sc-2324) overnight at 4 °C constantly shaking and incubated with the primary antibody in blocking solution (5% non-fat milk in TBST 1X). The following primary antibodies were used: rabbit polyclonal p42/p44 MAPK (Erk1/2) (1:3000, Cell Signaling, #9102) and mouse monoclonal phospho-p44/42 MAPK (Erk1/2) (Thr202/Tyr204) (1:1000, Cell Signaling, #9106). Following several washes in TBST 1X, membranes were incubated with anti-rabbit- (1:8000, Thermo Fisher, 31,460) and anti-mouse-HRP-conjugated secondary antibodies (1:3000, Thermo Fisher, 31,450). Immunoreactive proteins were detected by SuperSignal™ West Femto Maximum Sensitivity Substrate (Thermo Fisher, 34,095) according to the manufacturer’s instructions, and an Alliance Mini HD9 with Q9 Mini 18.02-SN software (Uvitec) was used for chemiluminescence detection. For densitometric analysis of specific protein bands the “free Band/Peak Quantification” tool of the Fiji software was used. Briefly, starting from the acquired gel images, rectangular ROI were drawn around the detected bands of interest (pERK, tERK and GAPDH) for each lane. Pixel intensity was then retrieved and measured for the selected ROIs. For each sample, values obtained for pERK and tERK were normalized to GAPHD levels. The ratio between the normalized pERK and tERK value was then calculated and reported.

### pERK levels detection and quantification via whole-mount immunohistochemistry

24 hpf zebrafish embryos were fixed in 4% PFA/PBS (Thermo Fisher, 28,908) for 3 h at RT. 6 hpf and 11 hpf embryos were fixed with 4% PFA/PBS-Triton 0.25% (Thermo Fisher, 28,908), overnight at 4 °C or 3 h at RT, respectively. Embryos were subsequentially washed in 0.8% PBS-Triton and stored in PBS 1X at + 4 °C. Immunostaining was adapted from a previously reported protocol (Myklatun, Lauri et al., 2018). Briefly, fixed samples were washed in PBS-Triton 0.8%. Only 11 hpf and 24 hpf embryos were then incubated with 150 mM Tris–HCl pH 9.0 for 5 min at RT and 15 min at 70 °C for antigen retrieval. Samples were permeabilized with 1 µg/ml proteinase K (Sigma-Aldrich, P2308) (2 min for 6 hpf and 11 hpf, 20 min for 24 hpf embryos) at RT, followed by post-fixation in 4% PFA/PBS for and incubation in blocking solution in 5% of normal goat serum (NGS), 1% of BSA, 1% DMSO in 0.8% PBS-Triton (20 min for 6 hpf and 12 hpf, for 2 h for 24 hpf embryos) at RT. The following primary antibodies were used: mouse monoclonal p44/42 MAPK (ERK, 1:250, Cell Signaling, 4696 S), rabbit polyclonal phospho-p44/42 MAPK (pERK, 1:250, Cell Signaling, 4695 S). The following secondary antibodies were used: goat anti-mouse Alexa Fluor 488 (1:600, Thermo Fisher, A11001) and goat anti-rabbit Alexa Fluor 633 (1:600, Thermo Fisher, A21070). The Stellaris 5 confocal microscope (Leica Microsystems) equipped with LAS X software v. 4.5 and an HC FLUOTAR L VISIR water immersion 25x/0.95 objective were used for z-stack image acquisition. *X,y,z* stacks of the embryos (mounted in 90% glycerol/PBS in case of 24 hpf and mounted in 1.5% low-melting agarose in PBS 1X for 6 hpf and 12 hpf embryos) were acquired sequentially in two channels to image pERK and tERK with 499 and 631 nm laser lines and emission range of 507–551 nm and 644–740 nm, respectively. Acquisitions of 6 hpf and 12 hpf embryos were obtained by scanning with a 512 × 512 format at 400 Hz and 5 μm *z*-step size and width and a standard digital zoom of 1. Brain acquisitions at 24 hpf were obtained by scanning with a 1024 × 1024 format at 200 Hz and 1.5 z-step size. Eye and tail acquisition at 24 hpf were obtained by scanning with a 512 × 512 format at 400 Hz and 5 *z*-step size. Relative pERK fluorescent intensity (Raw Integrated density of pERK normalized to Raw Integrated density of tERK) in defined ROI was retrieved from the acquired images by using the “Polygon” selection tool, “ROI manager” plug-ins in Fiji. Ratio of the intensity between pERK and tERK was calculated and reported.

### Statistical assessment and illustrations preparation

At least two researchers independently assessed all the datasets. Statistical comparisons were performed using unpaired One-tail Student’s t-tests and One-Way ANOVA with Dunnett’s and Holm-Sidak *post hoc* test as indicated in the relative figure legends using GraphPad Prism software v. 9.4.1. Data are represented as mean ± SEM (for parametric data) or median with interquartile range (for non-parametric data) and statistical significance was assumed by *p* < 0.05 (* *p* < 0.05, ** *p* < 0.01, *** *p* < 0.001, **** *p* < 0.0001). Exclusion criteria due to technical issues during imaging sessions are indicated in the respective Source data file, provided as Supplementary material. Outliers identified by the ROUT method (Q = 10%) in GraphPad Prism software were excluded. The schematic illustrations of this paper were generated using Illustrator (Adobe). Embryos, plate, tubes and western blot schematic images were obtained by BioRender.com.

## Results

### ERK activity changes are registered live in *teen* embryos upon positive and negative pharmacological modulation of RAS/MAPK signaling

**Fig. 1:**
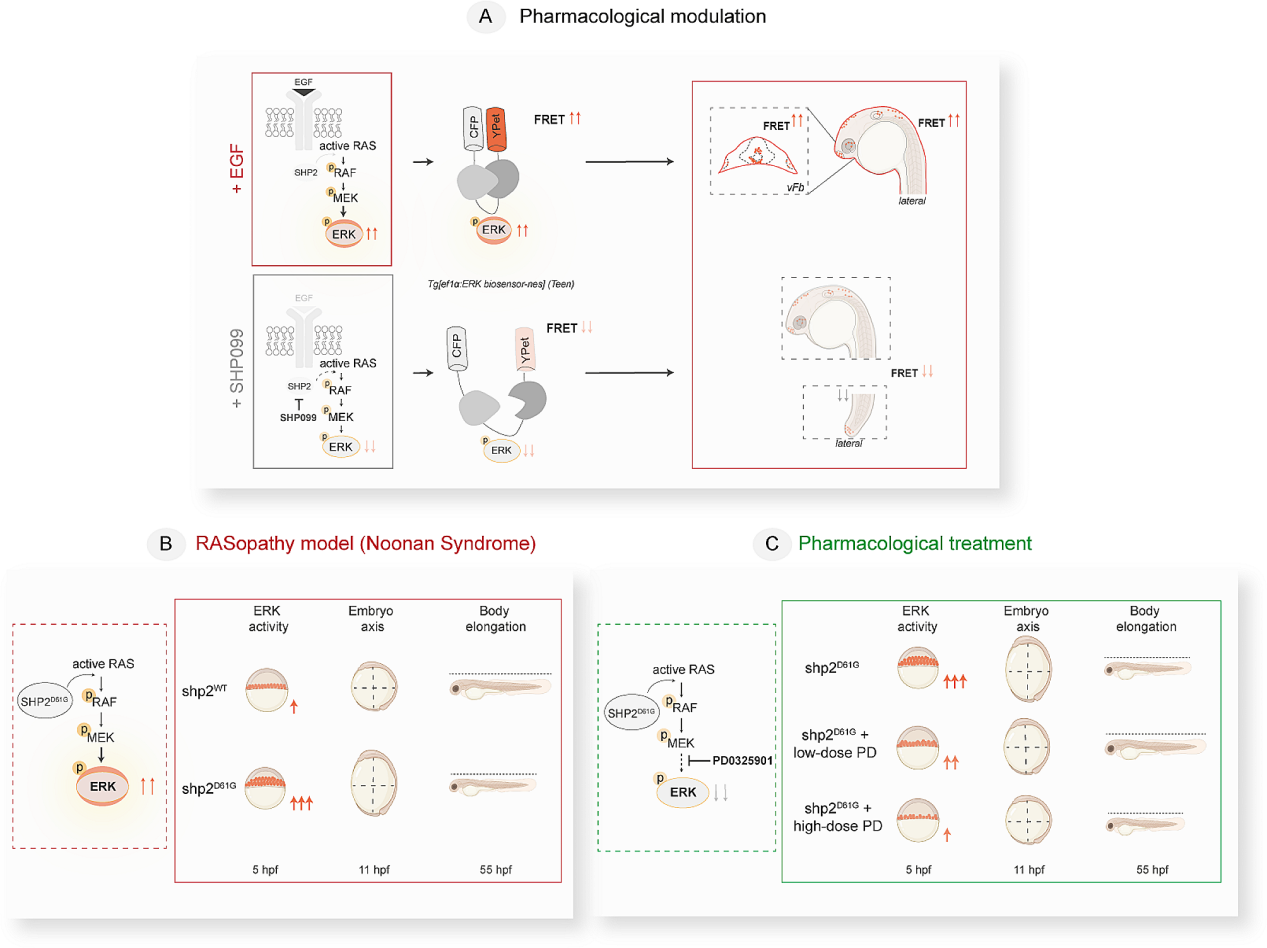
Schematics of the study design and outcome summarizing the main steps and outcomes of pharmacologically- and genetically induced RAS-MAPK pathway modulation assessed in *Teen* ERK reporter embryos. (**A**) Positive and negative modulation of RAS-MAPK signaling (via monitoring ERK activation) are obtained through pharmacological approach by acute stimulation with Epidermal growth factor, EGF, or prolonged exposure to the SPH2 inhibitor SHP099, respectively. ERK activation is assessed using the FRET-based ERK sensor *Teen*. In this study, an increase in the FRET signal in *Teen* embryos (due to elevated ERK activity) is visible in ventral forebrain upon EGF injection (upper panel). A decrease in FRET signal (due to reduced ERK activity) is visible in different brain domains and tail upon treatment with SHP099 (lower panel). (**B, C**) Genetic modulation of RAS-MAPK signaling in early embryos of a well-established Shp2^D61G^-NS zebrafish model (**B**) and partial rescue obtained by with low- (0.25 µM) and high-dose (1 µM) MEK inhibitor PD0325901 (PD) (**C**) are assessed by FRET in Teen embryos and precede onset of classical RASopathy morphological hallmarks (embryo axis and body length, validated at 11 hpf and 55 hpf, respectively). pERK: phosphorylated ERK

RASopathies are largely caused by mutations affecting signal transducers and modulators operating at various levels along the RAS-MAPK signaling pathway resulting in sustained ERK activity. The generalized upregulation of this signaling cascade during embryogenesis reflects the multisystem involvement of these disorders. Therefore, we first set out to assess the possibility of registering a non-physiological FRET signal increase obtained by acute stimulation with a growth factor able to activate signaling through this cascade (Fig. 1A, upper schematics); then we assessed ERK activity registration in the presence and absence of a negative signal modulation driven by a potent MEKi (Fig. 1A lower schematics). Only once we determined that positive and negative signal modulation could be recorded in control fish, we assessed the NS zebrafish model and the ability of low-dose MEKi to rescue aberrant ERK activity and morphological disease features (Fig. 1B,C).

**Fig. 2:**
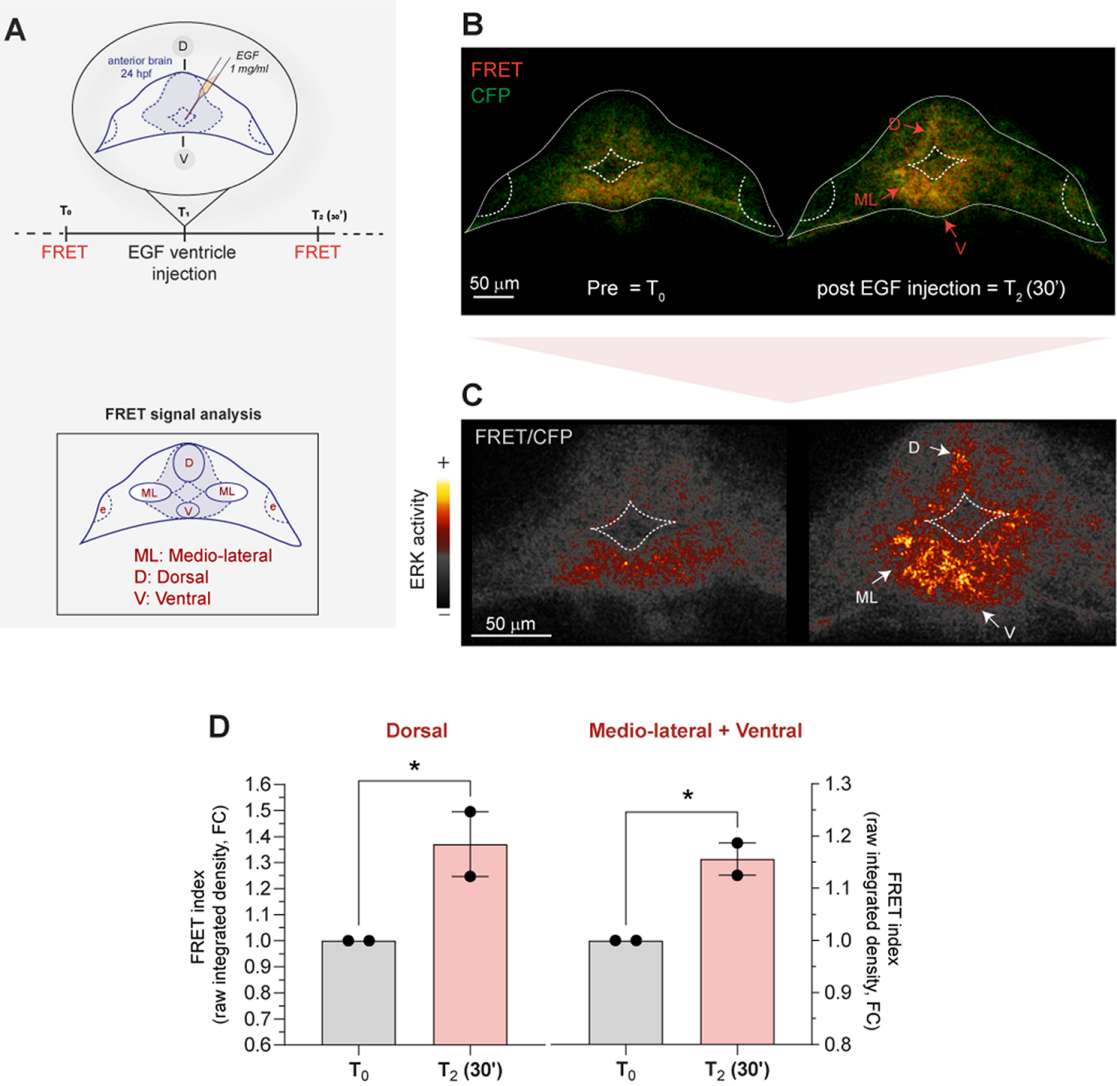
Increase in ERK activity is observed by spectral unmixing-FRET in the forebrain of live *Teen* embryos upon EGF ventricle injection. (**A**) The schematics depicts the experimental approach used to stimulate local pERK increase (ERK activity) within the anterior brain upon injection of rat EGF within the forebrain ventricle in live 24 hpf fish. FRET imaging using spectral unmixing mode was performed before (T0) and after T2 (30’) injection (T1). The lower schematics indicates the FRET signal analysis performed in various regions of interest around the ventricle, as indicated in the legend. (**B**) Representative sum-intensity projections of confocal *x,y,z, λ* live scans obtained by spectral unmixing from two embryos showing signal relative to FRET (red) and CFP (donor, green) before (pre) and 30 min after EGF injection (pre and post EGF, respectively). Red arrows mark FRET signal increase. D: dorsal, V: ventral, ML: medio-lateral domains. (**C**) Close ups of the sum-intensity projections of superficial *z*-layers of the ratiometric image (FRET/CFP) rendered with the “smart” LUT intensity scale and showing high FRET signal correlating to ERK activity (white arrows) in the dorsal (**D**), mediolateral (ML) and ventral (V) domains. (**D**) Bar graphs reporting the quantification and statistical support for FRET/CFP ratio (raw integrated density, arbitrary units, a.u. defined as “FRET index”) and expressed as fold change (FC) of treated *vs. *control fish. Dorsal: quantification in the dorsal domain. Mediolateral + Ventral: FC of the intensity detected in the medio-lateral (mean between right and left side) + the intensity of the ventral domain. Fold change (FC) data are expressed as mean ± SEM of two independent biological replicates, *n* = 2 embryos. Paired one-tailed t-test is used to assess statistical significance (* *p* < 0.05). Source data are provided as a Source Data file.

First, to obtain a positive control of signal modulation, we treated *Teen* reporter zebrafish 24 hpf embryos with high-dose recombinant EGF by direct microinjection within the anterior-most ventricles of the forebrain. Indeed, the diamond-shaped ventricles are visibly open during early stages (Lowery and Sive 2005) and readily accessible for microscope-guided manipulations, such as dye or drug injection (Fig. 2A). Moreover, this is a developmental domain with active RAS-MAPK signaling, as shown both in mice and in zebrafish embryos (Wong et al. 2019). We used EGF, given its known ability to induce RAS/MAPK activation and ERK phosphorylation (Kim and Choi 2010; Motta et al. 2020).

To acquire FRET information correlating to ERK activity in the *Teen* embryos we employed “multi-spectral imaging", upon donor excitation with 458 nm laser and collection of emission spectra between 460–570 nm. On the live acquisitions obtained we then performed “spectral unmixing”, employing the wizard provided within the confocal software, in order to perform donor and acceptor emission spectra decomposition (signal fingerprinting) of the CFP-YPet FRET pair.

We first ensured that the imaging conditions used for spectral unmixing were compatible with the correct development of the embryo from gastrulation to pharyngula-stage and that ERK activation observed using this method reproduced ERK activity patterns previously reported in embryos and using *Teen* sensor. As assessed by time lapse from late gastrulation to segmentation, *Teen* embryo developed normally (Supplementary video 1 and Supplementary Fig. 1). As reported (Sari et al. 2018; Wong et al. 2019), donor (CFP) signal was evident in the entire embryo and a relatively high FRET signal (active ERK) could be confirmed in regions previously reported for sustained ERK activity (i.e. margin of the animal pole in late gastrula), forming tail bud, forebrain, midbrain-hindbrain and segments in later embryo (Supplementary Fig. 1).

By comparing live spectral recording before (T0) and after (T2 = 30 min) EGF injection in 24 hpf embryos and quantifying the FRET/CFP signal intensity (FRET index), we were able to register increased ERK activity in tissue in the closed proximity of the anterior ventricle, especially within the ventral domain (Fig. 2B-D).

 If applied to a *x,y,z* acquisition of several z-planes and multiple wavelength bands, spectral unmixing is generally a lengthy procedure, requiring the live collection of many emission spectra, which limits fast imaging of ERK changes in a 4D (*x,y,z,t*). To validate the increased EGF-induced ERK activity we also employed bath-treatment of high-dose EGF in early embryos during somitogenesis ($\sim$  15 hpf and 20-24 hpf) and by simple scanning-based imaging mode using donor excitation at 458 nm and separated single emission windows for donor and acceptor analyzed in post-imaging ratiometric quantification. Conveniently, during these early phases a stratified epidermis forming a barrier between superficial and deeper layers is not yet formed (Li et al. 2011; Sampedro et al. 2018). In bath-treated embryos, we observed a global increase of ERK activity in known domains occurring already within 10 min from the start of the EGF treatment (Supplementary video 2 and Supplementary Fig. 2). The increased signal observed in both live FRET data, including the active ventral forebrain domain, was validated by standard whole-mount immunofluorescence performed against phosphorylated ERK (pERK) and normalized by total ERK (tERK) on embryos treated with the same concentration and timing of EGF (Supplementary Fig. 3A). The data were consistent also with western blot analysis corroborating increased levels of pERK from whole-embryo protein extracts upon 10 min of treatment (Supplementary Fig. 3B).

Next, calculation of the FRET index by spectral unmixing was also able to capture a global reduction of the signal correlating to ERK activity in live 24 hpf embryos obtained by signal inhibition, via the specific SHP2 inhibitor SHP099, compared to siblings treated with DMSO as control vehicle (Fig. 1A, lower schematics and Fig. 3A,B). A significantly reduced signal was evident when we performed ratiometric quantification of the signal in spatially distinct regions normally associated with high RAS-MAPK signaling (Fig. 3B,B’). We further validated the global reduction of the pERK signal observed by spectral unmixing in *Teen* embryos by western blot analysis from whole-embryo protein extracts of siblings treated with the same protocol (Supplementary Fig. 4).

Fig. 3Decreased ERK activity in live and fixe

**Figure:**
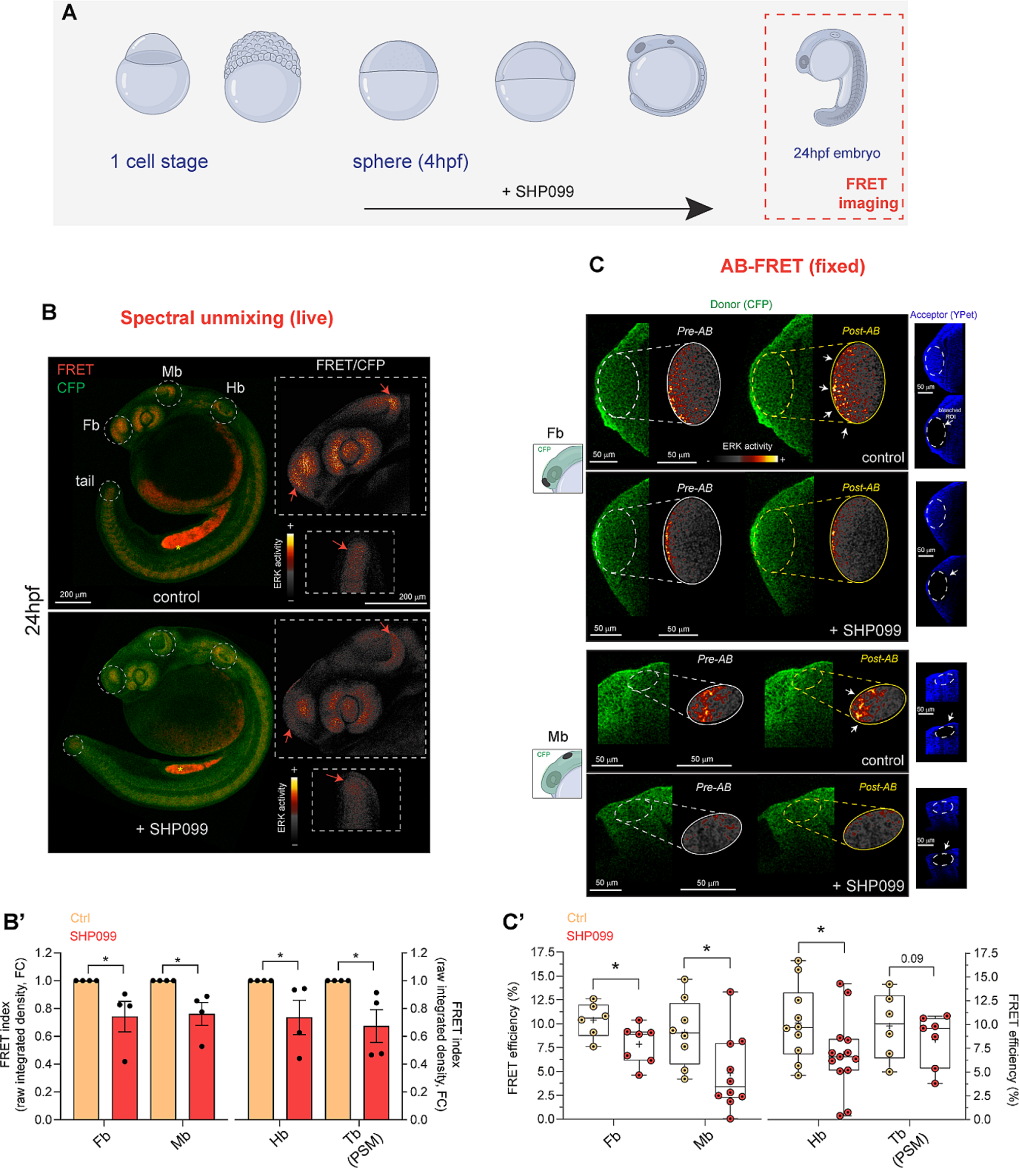
Decreased ERK activity in live and fixed Teen embryos upon prolonged SHP099 exposure is captured by spectral unmixing- and AB-FRET. (**A**) Schematics depicting the prolonged treatment with the Shp2 inhibitor SHP099 during zebrafish development from sphere stahe till 24 hpf (red square), stage employed in FRET imaging. (**B**) Representative sum-intensity projections of confocal *x,y,λ,z *live scans obtained by spectral unmixing from four embryos (treated either with DMSO vehicle control or with SHP099) showing FRET signal (red) and CFP (donor, green) emission. A FRET decrease, quantified in the different brain regions (Fb: forebrain, Mb: midbrain, Hb: hindbrain) and tail region (tailbud presomitic mesoderm, Tb PSM), is marked by dashed white circles and indicated by red arrows in insets, showing close-ups of the sum-intensity projections for the ratiometric image (FRET/CFP). Ratiometric images are rendered with the “smart” LUT intensity scale showing FRET signal correlating to ERK activity. (**B’**) Bar graphs reporting the quantification and statistical support for FRET/CFP ratio (raw integrated density, arbitrary units, a.u. defined as “FRET index”) and expressed as fold change (FC) of treated vs. control fish. Data are expressed as mean ± SEM of four independent biological replicates, n of embryos = 4. (**C**) Representative AB-FRET images before (Pre-AB panel) and after (Post-AB panel) AB-FRET. Acceptor bleaching in various regions (ROI 1, forebrain, Fb; ROI2, midbrain, Mb) is outlined by white dotted ellipses. In the schematics on the left ROI 1 and 2 are indicated with a black ellipse and shown on the right panel (Acceptor YPet, white arrows). Fb and Mb regions are sampled from the same embryo. (**C’**) The graph reports the FRET efficiency (E%) expressed as median with interquartile range from AB-FRET data for Fb and Mb (left panel) and Hb and Tb (PSM, right panel). In B’ and C’ one-tail t-test is used to assess statistical significance (*p < 0.05). For Control fish n  = 6, 8, 10, 6 (Fb, Mb, Hb and Tb, respectively). For SHP099-treated fish n  = 7, 10, 13, 7 (Fb, Mb, Hb and Tb, respectively). Source data are provided as a Source Data file.

The experiments showed that acute treatment of developing embryos with EGF and persistent treatment with the specific SHP2 inhibitor from early gastrula ( 4 hpf) to pharyngula stage (24 hpf) effectively activate and reduce ERK activation, respectively, in known domains characterized by high MAPK signaling, and that such modulation can be efficiently captured with live FRET imaging.

Signal changes assessed by western blot were marked and the relative change magnitude (effect size) was higher compared to FRET, likely depending on the limited dynamic range of the CFP-YFP pair (Lam et al. 2012). Nevertheless, by use of spectral unmixing and specific modulatory drugs, these results confirmed and broadened existing evidence that the protocols used for live FRET imaging in *Teen* embryos can be used to observe non-physiologically positive and negative modulation of ERK activity acting on the RAS/MAPK signaling in live fish.

### Acceptor photobleaching (AB)-FRET can detect decreased ERK activity mediated by pharmacological signal inhibition in fixed *teen* fish specimens

Next, we assessed the versatility of the *Teen* reporter to other FRET imaging protocols, that could broaden the application of this zebrafish ERK biosensor. We asked whether the ability to detect pharmacologically-induced changes of ERK activity observed in live tissue by FRET-dependent YPet acceptor emission measurements could be reproduced by deriving directly the energy transfer efficiency (E) from CFP Donor (D) to the YPet Acceptor (A). As introduced, this value correlates directly with the distance between D and A (R_DA_) and thereby, in *Teen* sensor, with ERK activity (Algar et al. 2019). To this aim we used the Acceptor photobleaching (AB)- FRET protocol where E can be estimated by measuring the fluorescence emitted by D and A before and after performing Acceptor Bleaching (AB). Because of the bleaching step and hence the incompatibility of AB-FRET for live acquisition, we turned to fixed samples.

We fixed a group of 24 hpf *Teen* fish which underwent the same SHP099 treatment protocol that produced potent signal inhibition in the live experiments. We limited the AB procedure mainly to representative regions of interest known for high RAS-MAPK signaling (various brain domains and the presomitic mesoderm, PSM of the growing tail bud region, Tb). In these domains, we could effectively derive a decrease in FRET E (%) upon SHP099 chronic treatment, with a stronger effect in the brain (Fig. 3A,C,C’). Increased distance between donor and acceptor in SHP099-treated embryos was observed when R_DA_ was retrieved from the efficiency data, which was significant for anterior brain regions (Supplementary Fig. 5). Overall, the data indicate that AB-FRET protocol applied to fixed *Teen* embryos can also be used to detect strong modulation of the signal.

### Increased ERK activity can be recorded by FRET in *teen* embryos expressing the NS-causing Shp2^D61G^ allele before the onset of quantifiable morphological defects

Having obtained FRET data proving effective detection of pharmacologically-induced positive and negative signal modulation in both live and fixed *Teen* embryos, we next tested the suitability of the approach to detect early signaling impairment caused by a recurrent pathogenic SHP2 amino acid substitution, D61G, causing NS, which determines increased ERK activation (Bonetti et al. 2014a; Zhu et al. 2020) as well as recognizable morphological defects occurring during embryogenesis (Bonetti et al. 2014a; Jindal et al. 2017; Bobone et al. 2021; Solman et al. 2022), typical of NS disease fish models (Supplementary Table 1). Therefore, we turned our analysis to an established zebrafish RASopathy model obtained by overexpressing Shp2 carrying the NS-causing D61G substitution in *Teen* embryos. Overexpression of wild-type Shp2 can induce a certain degree of phenotype in mice models (Hu et al. 2017). A significant effect of was not observed in fish injected with Shp2^WT^ compared to not injected control (Bonetti et al. 2014a; Paardekooper Overman et al. 2014; Bobone et al. 2021) (Supplementary Table 1) which supports the use of Shp2^WT^ as an internal control of Shp2 overexpression in fish microinjection experiments. Indeed, a clear pathological impact on morphological development of the mutant form (Shp2^D61G^), compared to overexpression of Shp2^WT^, can be scored in zebrafish models (Bonetti et al. 2014a; Paardekooper Overman et al. 2014; Jindal et al. 2017; Bobone et al. 2021). Therefore, we decided to use embryos overexpressing the WT protein as controls, and assessed the sensitivity of the FRET protocol in discriminating pathogenic signaling changes occurring upon Shp2^D61G^ compared to Shp2^WT^.

**Fig. 4:**
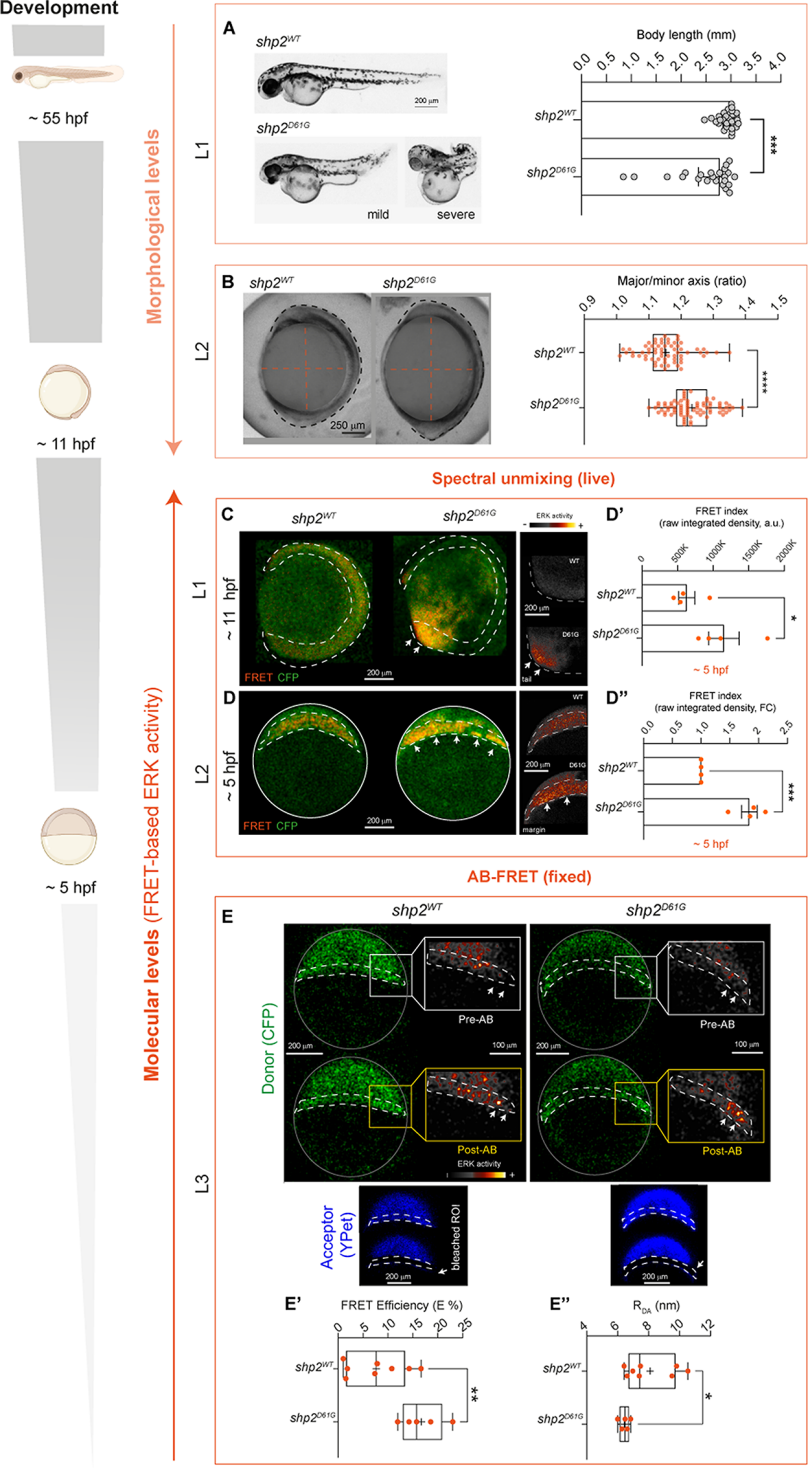
Increased ERK signal measured by spectral unmixing and AB-FRET in Shp2^D61G^ zebrafish mutants showing morphological defects. (**A**) Representative bright-field micrographs and graphs showing body length measurements of embryos overexpressing the WT and mutant (D61G) form of Shp2 at 55 hpf. Non-parametric Mann Whitney test is used to assess the statistical significance (*** p < 0.001). N = 28 and 23 (Shp2^WT^ and Shp2^D61G^ respectively). Data are expressed as median with interquartile range. (**B**) Representative bright-field micrographs of mutant embryos at early segmentation stage ( 11 hpf) compared to control fish (expressing Shp2^WT^). Major and minor axes defects are visible, outlined by a dashed orange lines and by the quantification of major/minor axis ratio. The box plot with the median (middle line), 25th–75th percentiles (box), and min–max values (whiskers) shows the quantification of major/minor axis ratios. One-tail Student t-test is used to assess the statistical significance (**** p < 0.0001). N = 77 and 64 (Shp2WT and Shp2D61G respectively). (**C, D**) Single plane images of confocal *x,y,z,λ,t *and sum-intensity projections of confocal *x,y,λ,z live* scans (**C** and **D**, respectively) obtained by spectral unmixing-FRET of embryos at 11 hpf (**C**) and 5 hpf (**D**) expressing Shp2^WT^ and Shp2^D61G^ and showing FRET (red) and CFP (donor, green) signals. Increase in ERK activity (FRET channel) in the tail bud (PSM) (**C**) and in the margin of the animal pole (**D**) of zebrafish embryos is indicated by white arrows. A dashed white line outlines the developing embryo (C) and the margin (**D**). Close-ups on the right rendered with “Smart” LUT in Fiji show increased signal in the tail (**C**) and margin (**D**) regions. (**D’, D’’**) Bar graphs reporting the quantification and statistical support for FRET/CFP ratio (raw integrated density, arbitrary units, a.u. defined as “FRET index”) and expressed as raw values (**D**’) as fold change (FC) of Shp2^D61G^*vs.* Shp2^WT^ (**D’’**). Data are expressed as mean ± SEM. One-tail t-test is used to assess the statistical significance (* *p* < 0.05, *** *p* < 0.001)). N of embryos = 4 (Shp2WT and Shp2D61G). (**E**) Representative confocal images (single plane) showing donor (CFP, green) before (Donor-Pre) and after (Donor– Post) AB-FRET for embryos expressing Shp2^WT^ and mutants expressing Shp2^D61G^. A dashed white line indicates the animal pole margin targeted for Acceptor Bleaching (AB). For each condition, insets of the right show close-ups on the donor (CFP) at the margin region before and after AB (pre- and post-, respectively) rendered with “Smart” LUT in Fiji. Bright pixels showing FRET signal increase upon AB are indicated by white arrows. Embryos are outlined by a continuous white line. (**E’-E’’**) Box plot with median (middle line), 25th–75th percentiles (box), and min–max values (whiskers) showing the quantification of AB-FRET efficiency (E %, E’) and RDA values (nm, E’’) in the margin of zebrafish 5hpf embryos overexpressing WT and mutant (D61G) Shp2. One-tailed t-test is used to assess the statistical significance (* *p* < 0.05, ** *p* < 0.01). N = 8 and 5 (Shp2WT and Shp2D61G respectively). L1-L3: different analysis levels for both morphological and molecular assessments. Source data are provided as a Source Data file.

First, we confirmed the characteristic shortening of the embryos accompanied by edema in hatched Shp2^D61G^ expressing fish (, 55 hpf*morphological level 1*, L1, Fig. 4A), which was anticipated by impaired axes establishment quantifiable at the end of gastrulation, when head and tailbuds begin to be visible and somitogenesis starts (11hpf, *morphological level 2*, Fig. 4B). Next, we examined by spectral unmixing one of the Shp2^D61G^ mutant *Teen* embryos exhibiting an aberrantly elongated major axis at this stage and noted a visible increase in FRET signal compared to the control fish expressing Shp2^WT^ (*molecular level 1, L1*, Fig. 4C). This was especially clear in the tail region (right insets in Fig. 4C).

We set out to assess whether an increased FRET signal could set apart Shp2^D61G^ activity on RAS-MAPK signaling already at around 5 hpf (approximately 40% epiboly, *molecular level 2, L2*, Fig. 4D). At these early stages, control and mutant embryos usually appear morphologically indistinguishable, and therefore early genotype-phenotype correlation which might speed up and simplify validation and drug screening is difficult. We specifically investigated the margin region of the animal pole where high FGF-induced ERK activity controls morphogenic gastrulation movements (Krens et al. 2008b; van Boxtel et al. 2018; Wong et al. 2019) and which is well represented in *Teen* sensors (Wong et al. 2019). Indeed, despite no morphological changes were visible in early gastrulae, we documented an increased signal in live Shp2^D61G^-expressing gastrulae by measuring spectral unmixing-derived FRET index (Figs. 1B and 4D-D’’). The live data obtained offer a first indication that an early molecular alteration at the level of ERK activity due to constitutively active Shp2 could potentially be detected in *Teen* NS mutant gastrulae by live FRET.

Given the success obtained with applying AB-FRET in SHP099-treated embryos, we investigated further this possibility and set out to examine directly the entity of transfer efficiency (E) by attempting AB in the margin region of the animal pole of fixed gastrulae. This additional experiment with a complementary FRET technique corroborated the occurrence of a detectable and statistically significant signal increase in a different clutch of NS mutant embryos (*molecular level 3, L3*, Fig. 4E,E’). The increase in E in NS mutants at 5 hpf proportionally correlated with a decrease of the derived R_DA_ (Fig. 4E’’ and Supplementary Fig. 6 ) and therefore with increased ERK activity.

### Dose-dependent reduction of pathogenic ERK activity is captured early in *teen* embryos treated with MEK inhibitor and predicts partial morphological rescue

Given that short window treatment with low-dose MEKi can partially rescue some morphological phenotypes in fish models of NS caused by Shp2^D61G^ (Bonetti et al. 2014a), we next asked whether spectral unmixing- FRET could detect signs of moderately reduced ERK activity supposedly obtained by MEKi already in early NS gastrulae. We used a high-dose (1 µΜ) of the MEKi PD0325901 as a strong control of the negative modulation on the signal. Indeed, by employing such a high-dose MEKi on control gastrulae (not injected) and spectral FRET imaging we detected a marked reduction of ERK activity (Supplementary Fig. 7), validating what was previously shown biochemically by van Boxtel et al. (van Boxtel et al. 2018) and in *Teen* sensors by Wong et al. (Wong et al. 2019).

Because of successful reports on prolonged treatment specifically with low-dose MEKi PD0325901 in another RASopathy model clinically related to NS, the cardio-facio-cutaneous syndrome (CFCS) caused by BRAF^Q257R^ (Anastasaki et al. 2012), we first tested whether longer treatments with low-dose MEKi could rescue body axis defects also due to expression of the NS-associated Shp2^D61G^.

Hence, we treated embryos with 0.25 µM or 1 µM PD0325901 from 4 hpf to the desired stage, employing long and short treatment windows and we first verified the occurrence of morphological rescue of major RASopathy traits, compared to fish treated with control vehicle (DMSO).

**Fig. 5:**
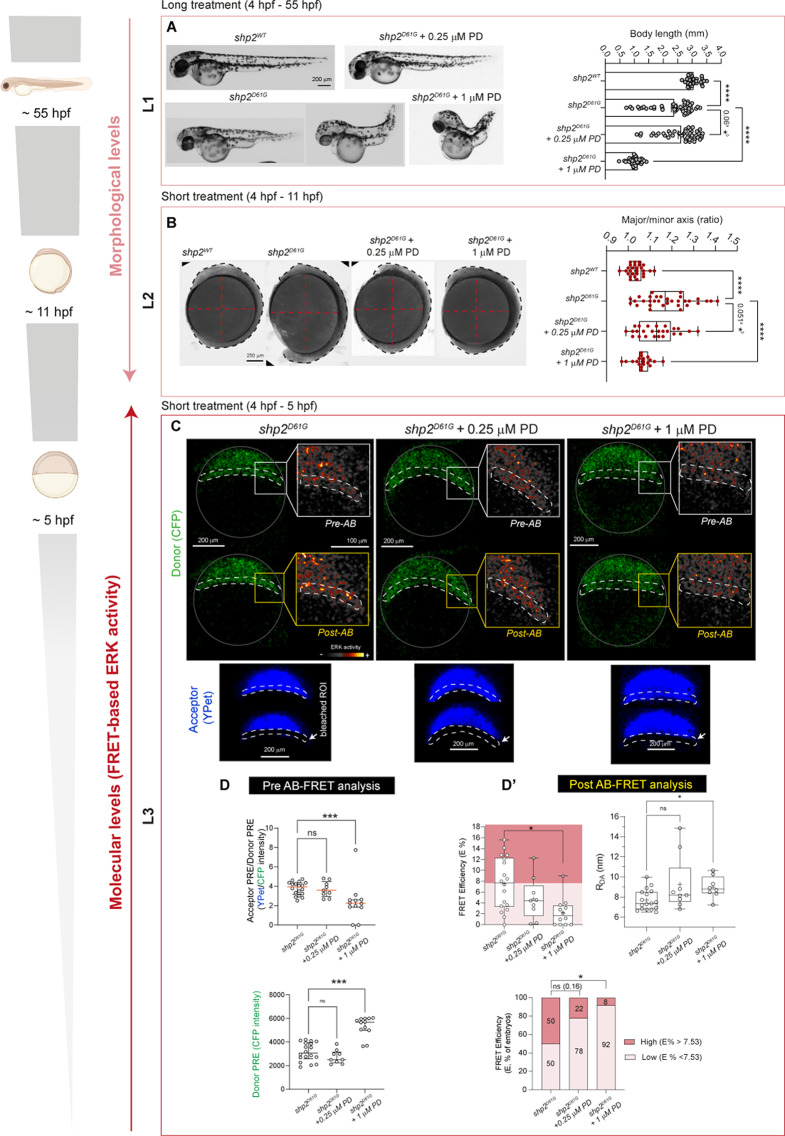
Reduced ERK activity measured by spectral unmixing and AB-FRET in zebrafish Shp2^D61G^ NS-causing mutants exhibiting morphological defects upon low- and high-dose MEK inhibitor treatment. (**A**) Representative bright-field micrographs of hatched zebrafish embryos expressing Shp2^WT^, Shp2^D61G^ treated with DMSO vehicle control (Shp2^D61G^) or with 0.25 µM and 1 µM PD0325901 (PD) since 4 hpf. The bar graph on the right shows body length measurements of zebrafish embryos expressing Shp2^D61G^ and the rescue obtained by low-dose and high-dose PD0325901 treatment. One-way ANOVA with Dunnett’s (^a^ 0.06, **** *p* < 0.0001) and Holm-Sidak (only for low-dose PD0325901 experimental group, ^b^ * *p* < 0.05) *post hoc* test is used to assess statistical significance after outliers’ removal (ROUT method Q = 1%). *N* = 31, 50, 43 and 44 (Shp2^WT^, Shp2^D61G^ - or + 0.25 µM PD and 1 µM PD respectively). (**B**) Representative bright-field micrographs of mutant embryos at early segmentation stage treated either with DMSO vehicle control (Shp2^D61G^) or with 0.25 µM and 1 µM PD compared to control fish (expressing Shp2^WT^). Major and minor axes defects are visible, outlined by a dashed red line, embryos are outlined by a dashed black line. Quantification of major/minor axis ratio is shown by the box plot with median (middle line), 25th–75th percentiles (box), and min–max values (whiskers). One-way ANOVA with Dunnett’s (^a^ 0.051, * *p* < 0.05, **** *p* < 0.0001) and Holm-Sidak (only for low-dose PD0325901 experimental group, ^b^ * *p* < 0.05) *post hoc* test are used to assess the statistical significance after outliers’ removal (ROUT method Q = 1%). Data are expressed as mean ± SEM of two independent biological replicates. *N* = 27, 32, 25 and 19 (Shp2^WT^, Shp2^D61G^ - or + 0.25 µM and 1 µM PD, respectively). (**C**) Representative confocal images (single plane) showing donor (CFP, green) before (Donor-Pre) and after (Donor–Post) AB-FRET for mutants treated with DMSO vehicle control (Shp2^D61G^) or with the PD0325901 (Shp2^D61G^ + 0.25 µM and 1 µM PD). A dashed white line indicates the animal pole margin targeted for Acceptor Bleaching (AB). For each condition, insets on the right show close ups on the donor (CFP) at the margin region before and after AB (pre- and post-) rendered with “Smart” LUT in Fiji. The acceptor (YPet, blue) is shown in the small inset below before and after bleaching of the margin region (dashed white line and arrow). Embryos are outlined by a continuous white line. (**D**) Quantification of signal intensity before AB-FRET. The upper and lower scatter plots (Median and interquartile range) show Acceptor/Donor (YPet, CFP) and CFP signal intensity, respectively. *N* = 18, 9 and 12 (shp2^D61G^, Shp2^D61G^ + 0.25 µM PD and Shp2^D61G^ + 1 µM, respectively). A marked reduction in the FRET (and reduced Donor quenching) is observed in NS mutants treated with 1 µM PD. Kruskal-Wallis with Dunn’s *post hoc* test is used to assess statistical significance (* *p* < 0.05). Data are expressed as median with interquartile range. N of embryos = 18, 9 and 12 (Shp2^D61G^, Shp2^D61G^ + 0.25 µM PD and Shp2^D61G^ + 1 µM, respectively). (**D’**) AB-FRET data quantification represented by the box plot with median (middle line), 25th–75th percentiles (box), and min–max values (whiskers) and showing the AB-FRET efficiency (E %) and RDA values (nm, right inset) in the margin of mutant embryos (Shp2^D61G^) treated with DMSO vehicle control or low and high-dose of PD0325901 (Shp2^D61G^ + 0.25 µM PD or 1 µM PD, respectively). One-way ANOVA with Dunnett’s *post hoc* test is used to assess the statistical significance (* *p* < 0.05). For FRET efficiency (**E**) dataset, n = 18, 9 and 12 (Shp2^D61G^, Shp2^D61G^ + 0.25 µM PD and Shp2^D61G^ + 1 µM, respectively). For R_DA_ dataset, values are excluded when E % values = 0 (exclusion criterion reported in the source data file), N = 17, 9 and 8 (Shp2^D61G^, Shp2^D61G^ + 0.25 µM PD and Shp2^D61G^ + 1 µM, respectively). The lower graph shows the percentage of embryos classified based on high or low values of AB-FRET-derived E (> or < 7.53, respectively). One-sided Chi-square’s test in a 2 × 2 contingency table (shp2^D61G^ vs. Shp2^D61G^ + 0.25 µM PD ns = not statistically significant, Shp2^D61G^ vs. Shp2^D61G^ + 1 µM PD * *p* < 0.05) is used to assess statistical significance. *N* = 18, 9, and 12 (Shp2^D61G^, Shp2^D61G^ + 0.25 µM PD and Shp2^D61G^ + 1 µM, respectively). L1-L3: different analysis levels (L1 and L2, morphological; L3 molecular). Source data are provided as a Source Data file

We found that constant long treatment till 55 hpf with low-dose MEK inhibitor partially rescued body shortening in hatching embryos (*morphological level 1*, *L1*, Fig. 5A). Likewise, a shorter treatment (between 4 hpf and 11 hpf ) was already able to rescue the aberrant ratio between the major and minor axes examined by “oval embryo assay” at early segmentation stage (*morphological level 2*, *L2*, Fig. 5B). Conversely, while 1 µM PD0325901 also induced rescue when used for a short time (between 4 hpf and 11 hpf), it negatively impacted embryo development if prolonged till hatching and even worsened body length phenotype of NS mutants (compared *L1* and *L2* for the high dose in Fig. 5A,B). Quantification of live FRET revealed a reduced ERK activity in embryos expressing Shp2^D61G^ treated with 0.25 µM PD0325901 that was visible from 6 h of treatment (between 4 hpf and 10 hpf and till 11,30 hpf, Supplementary Fig. 8A,B). The capacity of the MEKi to significantly block ERK activation in a dose-dependent manner during this crucial developmental window was further demonstrated by assessing pERK levels from whole-embryos protein extracts of NS fish. In agreement with the live imaging FRET imaging experiment and previous reports (Anastasaki et al. 2009, 2012; Bonetti et al. 2014a) treatment with low-dose yielded a statistically significant pERK/tERK reduction (Supplementary Fig. 8A,C).

Last, we assessed the suitability of AB-FRET, which permits testing a larger cohort and yielded convincing results in capturing increased ERK activity already in early NS gastrulae, to record dose-dependent, spatially restricted ERK modulation in early Shp2^D61G^ fish mutants upon a short treatment window (between 4 hpf and 5 hpf) (Fig. 5C-D’, *molecular level 3, L3*). Analysis of the signal registered at the margin region of early gastrulae before performing AB (Pre AB-FRET analysis) showed already a reduced spatial quenching of the donor in NS mutants treated with 1 µM PD0325901 for a couple of hours, examined by calculating YPet/CFP and CFP signal intensities (Fig. 5D). The values show a strong inhibition of the FRET phenomenon occurring within the *Teen* reporter at the animal pole, demonstrating a low residual ERK activity obtained by relatively short treatment with high-dose PD0325901.

**Fig. 6:**
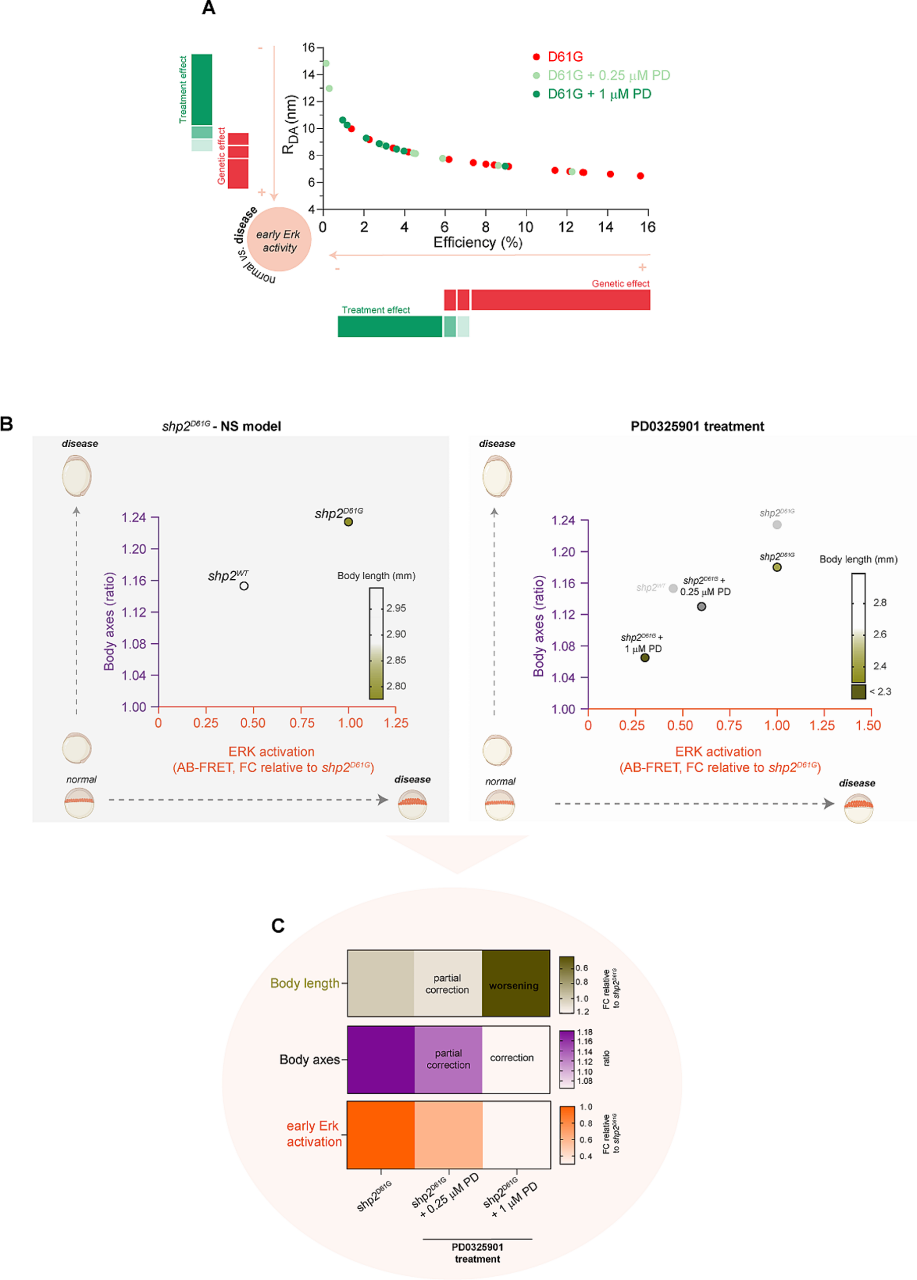
Molecular and morphological effect of the treatment with MEK inhibitor PD0325901 on zebrafish embryos overexpressing Shp2^D61G^ and showing developmental features modeling NS. (**A**) AB-FRET efficiency (E %, x) and RDA values (nm, y) calculated from AB-FRET on 5 hpf zebrafish embryos expressing mutant (D61G, red) Shp2 - and + treatment with low (0.25 µM, light green) and high (1 µM, dark green) doses of the MEK inhibitor PD0325901. High E values correlating with low R_DA_ values are schematically indicated by a red bar as the “genetic effect” relative to the Shp2^D61G^ allele. On the contrary low E values correlating with high R_DA_ values are indicated by a green bar as the “treatment effect” on the NS mutants due to short PD exposure. N of embryos = 18, 9, 12 (Shp2^D61G^, Shp2^D61G^ + 0.25 µM PD and Shp2^D61G^ + 1 µM, respectively). (**B**) Schematics and x,y graph showing the correlation between ERK activation (measured as AB-FRET E FC, x axis) and body axes (measured as ratio between major and minor axis, y axis) in zebrafish embryos expressing Shp2^D61G^ compared to control (Shp2^WT^) (left) or Shp2^D61G^ treated with DMSO vehicle control or with low- and high- PD doses (right). Modulations of the oval shape of the embryo measured at 11 (y axis) and of ERK activity measured by FRET already at 5 hpf (x axis) are depicted by a schematic illustration. A Heat map on the side of the graph indicates the severity of body shortening at 55hpf (dark bronze = severe). (**C**) Summary heat maps showing the correlation between ERK activity at 5hpf (orange, bottom graph, obtained by AB-FRET measurement), body axes morphology at 11hpf (violet, middle graph, major/minor axis ratio measurement) and embryo elongation at 55hpf (bronze, upper graph, body length measurement) in NS mutants expressing Shp2^D61G^ treated with DMSO vehicle control or with low (0.25 µM) and high (1 µM) PD0325901 doses. High ERK activity in NS mutants (expressing Shp2^D61G^) decreases in a dose-dependent manner upon short-time treatment with low and high PD0325901 doses. Accordingly, in a dose-dependent effect partial or complete morphological correction is observed at the level of body axis measured at 11 hpf. Toxicity with prolonged 1 µM PD0325901 treatment is shown for body length (worsening of the body length phenotype measured in embryos at 55 hpf)

The strong effect of high-dose MEKi in the animal pole margin region was further confirmed by analyzing the signal registered upon AB in the same embryos (Post AB-FRET analysis). In particular, comparing directly the estimated transfer E % and the R_DA_ obtained from measuring NS embryos treated with low-dose (0.25 µM) vs. high-dose (1 µM) MEK inhibitor, we documented a milder signal reduction at the margin region obtained by short treatment window with low-dose treatment (Fig. 5C,D’and Fig. 6A), a dose which was sufficient to induce partial morphological rescue if embryos were treated till completion of gastrulation. The AB-FRET data are consistent with the mild and strong reduction observed from whole-embryo extracts of fish treated with low and high MEKi dose, respectively, for a longer time window (Supplementary Fig. 8C).

Overall, when visualizing the whole collected data by plotting the measures of the earliest morphological sign observed in our NS model (body axes defect at 11 hpf ) *vs.* E % values retrieved from early AB-FRET experiments (5 hpf ) in a *x,y* graph, we can appreciate the pathogenic effect of the NS mutation (Shp2^D61G^*vs.* Shp2^WT^) correlating to aberrant ERK activation and differential impact of low- vs. high-dose MEK treatment on early signaling and embryo morphogenesis (Fig. 6B). From the overall data summary in Fig. 6A, B (right panel) and Fig. C, it appears also evident that the strong modulatory effect on ERK activity observed upon short treatment with 1 µM PD0325901 in gastrulae is able to rescue axes defects when treatment till gastrulation is performed. However, this treatment option worsens the body length defects in NS mutants if prolonged till embryos hatching, confirming the same toxicity observed upon prolonged treatment of other RASopathies fish models (Grzmil et al. 2007; Anastasaki et al. 2009, 2012).

Last, by employing whole-mount immunofluorescence against pERK in early gastrulae we could validate the data acquired by live- and AB-FRET and confirmed an increase in the relative levels of pERK (normalized to total ERK levels) at the margin of the animal pole in Shp2^D61G^ expressing fish, as well as an effective modulation by low and high-dose PD treatment in a dose-dependent manner (Supplementary Fig. 9).

## Discussion

The ease of transparent, rapidly developing zebrafish embryos as experimental animal model for rapid functional classification of genomic variants and search of mechanisms implicated in RASopathies is well established (Runtuwene et al. 2011; Bonetti et al. 2014a, b; Nakagama et al. 2020; Motta et al. 2021). With respect to the convenience of embryos-based screenings to test *off label* treatments, new drugs as well as therapeutic windows, zebrafish is unsurpassed among vertebrate models, also for RASopathies (Anastasaki et al. 2012; Patton et al. 2021; Bonetti et al. 2014a). However, current functional workflows in zebrafish applied to RASopathies are mainly based on morphological readouts appearing only in late embryos (*e.g.* body length reduction or craniofacial defects). The earliest morphological hallmark, namely an impairment of the embryonic axes, can be observed only once gastrulation is completed and segmentation begins (between 10 and 12 hpf). Moreover, monitoring of ERK activation status is currently determined mainly by static western blot analysis.

The establishment of recent reporter systems supposedly able to capture ERK activity fluctuations during embryogenesis offers the potential to expand this functional approach to RASopathies towards detecting early molecular signs that can speed up variants’ strengths and impact analysis as well as classification, following Patel et al. 2019 (Patel et al. 2019). These sensors can also serve as fast molecular readouts for drug testing in vivo.

With such potentials in mind, the pilot workflow presented here supported the suitability of one of the latest FRET-based ERK reporter system (*Teen* sensor) developed by Sari et al. (2018) to register positive and negative modulation of ERK activity in vivo, report early ERK activity changes, anticipating phenotype occurrence and low-dose pharmacological correction in a well-established RASopathy model.

Classical EKAR reporters are user-friendly intra-molecular FRET sensors using CFP and YFP fluorescent molecules and variants as donors and acceptors. This pair is ideal for FRET given the high quantum yield of the CFP and the high absorption ability of the YFP. Nevertheless, the known limited dynamic range of this type of sensors can result in poor detection of subtle but biologically relevant changes (Lam et al. 2012). Moreover, dynamic changes can be further attenuated in vivo (Nagai et al. 2004). The *Teen* biosensor is based on an optimized version of EKAR, called EKAREV, which employs ECFP and Ypet and a longer backbone (Komatsu et al. 2011). This improved version slightly betters the overall sensitivity of the sensor by rendering the FRET changes more dependent upon the actual distance between donor and acceptor, and thereby upon ERK activity. Despite not reporting massive signal changes, Sari et al. 2018 and Wong et al. 2019 successfully employed *Teen*, which recapitulated known spatial and temporal ERK dynamics during early development, somitogenesis and brain patterning. Moreover, they also showed the ability of the reporter to detect negative modulation mediated by high-dose MEKi, as previously demonstrated with biochemical approaches in fish (van Boxtel et al. 2018).

In this work, we showed that, despite the dynamic range limitation, a “spectral unmixing”-FRET protocol can capture significant ERK activation live in WT embryos upon RAS/MAPK cascade stimulation obtained by acute EGF exposure. Increased live ERK activity was observed also in the ventral forebrain, consistent with data in mice, pointing to the high susceptibility of this domain to RAS-MAPK signaling activation, likely under the local control of SHP2 (Gauthier et al. 2007; Ehrman et al. 2014). As demonstrated by mutant mice expressing constitutively active NS-causing SHP2^D61G^, this regulatory phosphatase probably plays a crucial role in balancing progenitors’ differentiation during cortex development, controlling gliogenesis and neurogenesis (Gauthier et al. 2007). Further studies would be necessary to assess whether a similar control is established in zebrafish anterior brain.

Live FRET imaging could also capture negative modulation of the signal that we obtained by inhibiting directly Shp2 via SHP099 or by high-dose MEKi treatment. Moreover, the employment of a different FRET protocol (AB-FRET) allowed us also to register potent ERK signal inhibition upon SHP099 on fixed specimens. Immunoblots from protein extracts of fish embryos treated with EGF or SHP099 confirmed the pERK signal modulations registered by FRET. Nevertheless, as expected based on the increased sensitivity of the technique and the poor performance of the FRET pairs in terms of dynamic range, immunoblot analysis showed a stronger signal modulation.

These pilot data corroborate that, despite the limited sensitivity, the Teen sensor in vivo allows to record positive and negative pharmacologically-induced ERK modulation with the use of FRET protocols on both live and fixed specimens.

Demonstrating the relevance of the EKAREV FRET-based ERK activity readout in whole embryos for human diseases, by using the validated live FRET and AB-FRET protocols here, we were able to register increased ERK activity in a transient NS zebrafish model generated by over-expression of the mRNA encoding Shp2^D61G^. Specifically, in the first step of our experimental workflow, we verified the occurrence of a subset of the morphological hallmarks normally used to score NS (and other RASopathies) from late to early embryos. Body elongation and axes morphogenesis defects (*morphological level 1 and level 2*) were confirmed in our model, consistently with the known role of RAS-MAPK signaling in paraxial mesoderm and gastrulation in various species (Delfini et al. 2005; Gervaise and Arur 2016; Hayashi and Ogura 2020).

During early segmentation stages, in which axes defects begin to be quantifiable, in vivo FRET imaging by “spectral unmixing” showed encouraging results in capturing spatially-restricted diseased NS-associated ERK activation (*molecular level 1*). Furthermore, the in vivo FRET index calculated from the signal detected already in early gastrulae demonstrated that, despite the discussed limited dynamic range, the technique might be sensitive enough to moderate/mild signs of ERK activity increases occurring in NS mutant embryos ahead of the start of head and tail morphogenesis (*molecular level 2*). Moreover, we additionally assessed and reported the positive performance of AB-FRET in documenting early ERK activity increase occurring in morphologically indistinguishable NS gastrulae (expressing Shp2^D61G^) compared to WT animals (expressing Shp2^WT^). The effect was clear when examining the margin of the animal pole, where active RAS-MAPK signaling is crucial during embryogenesis for epiboly and gastrulation movements (Krens et al. 2008a; Wong et al. 2019) (*molecular level 3*).

Altogether the experiments show the suitability of both live- and AB-based FRET for RASopathy signal investigation in vivo in fish models. The results demonstrate that Shp2^D61G^ causes an increased ERK activity early in the animal pole margin of gastrulating embryos, preceding the onset of discernable morphological defects.

Last, expanding on various reports of successful employment of mice and fish models (Anastasaki et al. 2009, 2012; Chen et al. 2010; Hernández-Porras et al. 2014; Inoue et al. 2014; Gelb et al. 2022; Hebron et al. 2022; Bonetti et al. 2014a) and together with the work by Sari et al. (2018) and Wong et al. (2019) we provide a ground assessment for the possible application of various FRET protocols in RASopathies fish models expressing *Teen* biosensor as molecular spatio-temporal readout in vivo to assess early effects of pharmacological interventions.

To this aim, the definition of effective concentrations and critical developmental window via the establishment of quantitative readouts, matched by spatial and temporal molecular information in animal models, is essential.

We used low-dose potent MEKi developed as a targeted pharmacological molecule for cancer treatment (Cheng and Tian 2017) that, given the mechanistic overlap, were proposed as a possible treatment in RASopathies (Andelfinger et al. 2019; Gelb et al. 2022) (Anastasaki et al. 2009, 2012; Bonetti et al. 2014a).

The results obtained by interrogating the morphological and molecular levels in our workflow showed that chronic treatment with low-dose PD0325901 can rescue partially body elongation defects caused by Shp2^D61G^. Of note, the partial morphological rescue obtained is matched by a moderate reduction in ERK activity registered in early embryos and preceding the morphological rescue. Together with additional evidence in other mice and fish RASopathies models, including RAF and SOS1-dependent NS mice models (Pagani et al. 2009; Chen et al. 2010; Holter et al. 2019), BRAF-dependent CFC and NS fish models (Anastasaki et al. 2009, 2012; Bonetti et al. 2014a) as well as a NS model in *Drosophila* (Oishi et al. 2006), and recent developments in FRET-based in vivo applications (Sari et al. 2018; Patel et al. 2019; Wong et al. 2019; Wilcockson et al. 2022), our data support the usefulness of animal models as well as reporter systems to advance pre-clinical applications in the field of RASopathies.

Furthermore, when comparing directly fixed NS mutant gastrulae treated with low- vs. high-dose MEK inhibitor by calculating the efficiency of energy transfer (E) and deriving the estimated relative distance between donor and acceptor (R_DA_), AB-FRET is able to show a dose-dependent spatially-restricted response in terms of ERK activity in very early (asymptomatic) fish. Immunofluorescence against the active form of ERK corroborated the occurrence of a dose-dependent decrease in ERK activation at the margin of the animal pole in early gastrulae upon PD0325901 treatment.

Importantly, despite the specificity of the PD0325901, the morphological analysis presented here also confirms the toxicity previously observed in WT fish and CFCS models with prolonged high-dose treatment (Grzmil et al. 2007; Anastasaki et al. 2009, 2012) pointing to the importance of the developmental time window and to restore a proper equilibrium of the RAS-MAPK pathway levels during embryogenesis.

By examining the utility of *Teen* sensor fish in the context of NS, this work demonstrates that early ERK activity fluctuations at the animal pole caused by genetic mutations, as well as a gradual signal modulation on diseased fish obtained by MEK inhibitors, precede and correlate with morphological changes. These fluctuations can be registered by using different FRET protocols already in early gastrulae just a few hours after birth, in both live and fixed specimens.

Certainly, the use of the zebrafish model and biosensor in vivo comes with the unique benefit of performing live recordings to map physio-pathological signaling fluctuations and immediate in vivo responses to drug treatments. Indeed, we demonstrated the usefulness of the *Teen* reporter in the context of RASopathies by using a non-invasive, non-disruptive multi-spectral imaging and unmixing. Conversely to AB-FRET, multi-spectral imaging is a reliable sensitized emission method to obtain accurate FRET measurements in live embryos with low phototoxicity (Dickinson et al. 2001; Zimmermann et al. 2002; Ecker et al. 2004; Gu et al. 2004). Nevertheless, AB-FRET modules (Zeug et al. 2012) offer the possibility to additionally test a larger cohort of fixed embryos in a relatively fast manner at an informative stage. Compared to 3D lambda scans (*x,y,z,λ*) used in multi-spectral imaging, AB-FRET is a more straightforward method because fast bleaching is performed only on* x,y* images and in defined ROIs. FRET Efficiency (E) values are directly provided (Patterson et al. 2000; Hennigan et al. 2009; Vogel et al. 2014) (Algar et al. 2019), without requiring data post-processing (conversely to multi-spectral imaging requiring a spectra unmixing step) nor ratiometric intensity analysis. Last, AB-FRET should be considered as a complementary approach to standard immunofluorescence methods. As an entirely optical method, AB-FRET is less labor-intensive, such that to obtain ERK activity estimates, it does not require special tissue preparation (such as permeabilization), staining steps with primary and secondary antibodies, *x,y,z* confocal scanning and post-imaging quantitative measurements.

## Conclusion

In conclusion, our work supports the use of FRET on live and fixed *Teen* ERK reporter fish as a whole-embryo molecular readout to map disease-associated as well as pharmacological-induced modulation of ERK correlates in RASopathy research. Future studies will be needed to confront dynamic and spatially-resolved fluctuations of ERK signaling in different RASopathies models and to expand critically of our pilot assessment on the suitability of the proposed protocol to examine alternative drugs targeting RAS-MAPK signaling now increasingly available. Continuous optimization of FRET pairs (Gohil et al. 2022) and of FRET imaging approaches (Zaza et al. 2023) will improve resolution and sensitivity, boosting further the potential of these types of bionsensor for pre-clinical applications.

### Electronic supplementary material

Below is the link to the electronic supplementary material.

Supplementary Video 1

Supplementary Video 2

Supplementary Material 2

Supplementary Table 1

Supplementary Material 1

## Data Availability

Raw uncropped blots and measurement data used for generating graphs in this study are provided as Supplementary Information. Confocal scans as well as the constructs generated in this study are made available by corresponding authors upon request.
