# Supplementary material for: Assessment of the FRET-based Teen sensor to monitor ERK activation changes preceding morphological defects in a RASopathy zebrafish model and phenotypic rescue by MEK inhibitor
Source: Mol Med. 2024 Apr 9;30:47. doi: 10.1186/s10020-024-00807-w (PMC11005195; doi:10.1186/s10020-024-00807-w)
Supplement: Supplementary file 4 — Supplementary Table 1 [file 10020_2024_807_MOESM4_ESM.pdf]

Morphological parameters	Target protein involved in the RASopathy model	Reference
Early convergence and extension defects , body length reduction, heart hypertrophy, cranio-facial defects	SHP2	Jopling C, van Geemen D, den Hertog J. Shp2 knockdown and Noonan/LEOPARD mutant Shp2-induced gastrulation defects. PLoS Genet. 2007 Dec;3(12):e225. doi: 10.1371/journal.pgen.0030225.
Convergence and extension defects, body length reduction, cranio-facial defects, increased pigment cell number (iridophores and melanophores), arrested neural crest progenitor differentiation and migration	SHP2	Stewart RA, Sanda T, Widlund HR, Zhu S, Swanson KD, Hurley AD, Bentires-Alj M, Fisher DE, Kontaridis MI, Look AT, Neel BG. Phosphatase-dependent and -independent functions of Shp2 in neural crest cells underlie LEOPARD syndrome pathogenesis. Dev Cell. 2010 May 18;18(5):750-62. doi: 10.1016/j.devcel.2010.03.009.
Body length reduction, cranio-facial defects, impaired heart function and morphogenesis	SHP2	Bonetti M, Rodriguez-Martinez V, Paardekooper Overman J, Overvoorde J, van Eekelen M, Jopling C, Hertog Jd. Distinct and overlapping functions of ptpn11 genes in Zebrafish development. PLoS One. 2014 Apr 15;9(4):e94884. doi: 10.1371/journal.pone.0094884. PMID: 24736444; PMCID: PMC3988099. Bonetti *et al.,* 2014
Oval shape, Body length reduction heart anomalies	SHP2	Bonetti M, Paardekooper Overman J, Tessadori F, Noël E, Bakkers J, den Hertog J. Noonan and LEOPARD syndrome Shp2 variants induce heart displacement defects in zebrafish. Development. 2014 May;141(9):1961-70. doi: 10.1242/dev.106310. Epub 2014 Apr 9. PMID: 24718990.
Oval shape	SHP2	Bobone S, Pannone L, Biondi B, Solman M, Flex E, Canale VC, Calligari P, De Faveri C, Gandini T, Quercioli A, Torini G, Venditti M, Lauri A, Fasano G, Hoeksma J, Santucci V, Cattani G, Bocedi A, Carpentieri G, Tirelli V, Sanchez M, Peggion C, Formaggio F, den Hertog J, Martinelli S, Bocchinfuso G, Tartaglia M, Stella L. Targeting Oncogenic Src Homology 2 Domain-Containing Phosphatase 2 (SHP2) by Inhibiting Its Protein-Protein Interactions. J Med Chem. 2021 Nov 11;64(21):15973-15990. doi: 10.1021/acs.jmedchem.1c01371.
Body length reduction, cranio-facial defects, heart anomalies	SHP2	Solman, M., Blokzijl-Franke, S., Piques, F., Yan, C., Yang, Q., Strullu, M., et al. (2022). Inflammatory response in hematopoietic stem and progenitor cells triggered by activating SHP2 mutations evokes blood defects. ELife 11, e73040. doi:10.7554/ELIFE.73040
Convergence and extension defects, body length reduction, cranio-facial defects, heart edema	SHP2	Paardekooper Overman J, Preisinger C, Prummel K, Bonetti M, Giansanti P, Heck A, den Hertog J. Phosphoproteomics-mediated identification of Fer kinase as a target of mutant Shp2 in Noonan and LEOPARD syndrome. PLoS One. 2014 Sep 3;9(9):e106682. doi: 10.1371/journal.pone.0106682. PMID: 25184253; PMCID: PMC4153654.
Oval shape, heart anomalies	MEK1 (MAPK kinase)	Jindal GA, Goyal Y, Yamaya K, Futran AS, Kountouridis I, Balgobin CA, Schüpbach T, Burdine RD, Shvartsman SY. In vivo severity ranking of Ras pathway mutations associated with developmental disorders. Proc Natl Acad Sci U S A. 2017 Jan 17;114(3):510-515. doi: 10.1073/pnas.1615651114.
Early convergence and extension defects, oval shape, body length reduction, heart anomalies and cranio-facial defects	BRAF	Anastasaki C, Estep AL, Marais R, Rauen KA, Patton EE. Kinase-activating and kinase-impaired cardio-facio-cutaneous syndrome alleles have activity during zebrafish development and are sensitive to small molecule inhibitors. Hum Mol Genet. 2009 Jul 15;18(14):2543-54. doi: 10.1093/hmg/ddp186.<br><br>Anastasaki C, Rauen KA, Patton EE. Continual low-level MEK inhibition ameliorates cardio-facio-cutaneous phenotypes in zebrafish. Dis Model Mech. 2012 Jul;5(4):546-52. doi: 10.1242/dmm.008672. Epub 2012 Feb 2. PMID: 22301711; PMCID: PMC3380717.

Supplementary table 1: Overview of the morphological parameters commonly assessed in representative zebrafish RASopathies caused by mutations in key genes belonging to the RAS/MAPK signaling pathway.
