# Supplementary material for: Assessment of the FRET-based Teen sensor to monitor ERK activation changes preceding morphological defects in a RASopathy zebrafish model and phenotypic rescue by MEK inhibitor
Source: Mol Med. 2024 Apr 9;30:47. doi: 10.1186/s10020-024-00807-w (PMC11005195; doi:10.1186/s10020-024-00807-w)
Supplement: Supplementary file 5 — Supplementary Material 1 [file 10020_2024_807_MOESM5_ESM.docx]

**SUPPLEMENTARY MATERIAL**

**Supplementary Figure 1:** *In vivo* time lapse in *Teen* embryos obtained by spectral unmixing-FRET protocol reporting high ERK activity in zebrafish embryos from late gastrulation till segmentation stage.

**Supplementary Figure 2:** Live reporting of increased ERK activity captured in zebrafish embryos at segmentation stage expressing the FRET-based ERK sensor *Teen* upon EGF stimulation.

**Supplementary Figure 3:** Increased pERK levels upon acute treatment of wild-type embryos with EGF is validated by immunofluorescence and western blot analysis.

**Supplementary Figure 4:** Decreased pERK signal upon chronic exposure with SHP099 in zebrafish embryos is validated by western blot analysis

**Supplementary Figure 5:** Increased R_DA_ values calculated from AB-FRET data of *Teen* embryos treated with SHP099.

**Supplementary Figure 6.** Visual correlation between AB-FRET efficiency (E %) and derived R_DA_ values in 5 hpf zebrafish *Teen* embryos over-expressing WT and mutant (D61G) Shp2*.*

**Supplementary Figure 7**: Live FRET of control gastrula (7 hpf) before and after treatment with high-dose PD.

**Supplementary Figure 8:** Decreased ERK signal in NS embryos (expressing Shp2^D61G^) treated with low-dose MEK inhibitor is measured by spectral unmixing-FRET during segmentation stages

**Supplementary Figure 9:** Immunofluorescence validation of the effect of low- and high-dose PD treatment in early NS fish models.

**Supplementary Movie 1:** *In vivo* time lapse movie obtained by spectral unmixing-FRET module (xyzλt) reporting ERK activity map of a zebrafish control *Teen* embryo from late gastrulation (~ 9 hpf) till late segmentation stage (~ 18 hpf) with a time interval of 30 min.

**Supplementary Movie 2:** *In vivo* time lapse movie obtained by xyzλt scanning of CFP and FRET signals from a *Teen* embryo before and after EGF bath stimulation.

**Source data**

Source data containing raw uncropped blots and raw data relative to main/supplementary figures.


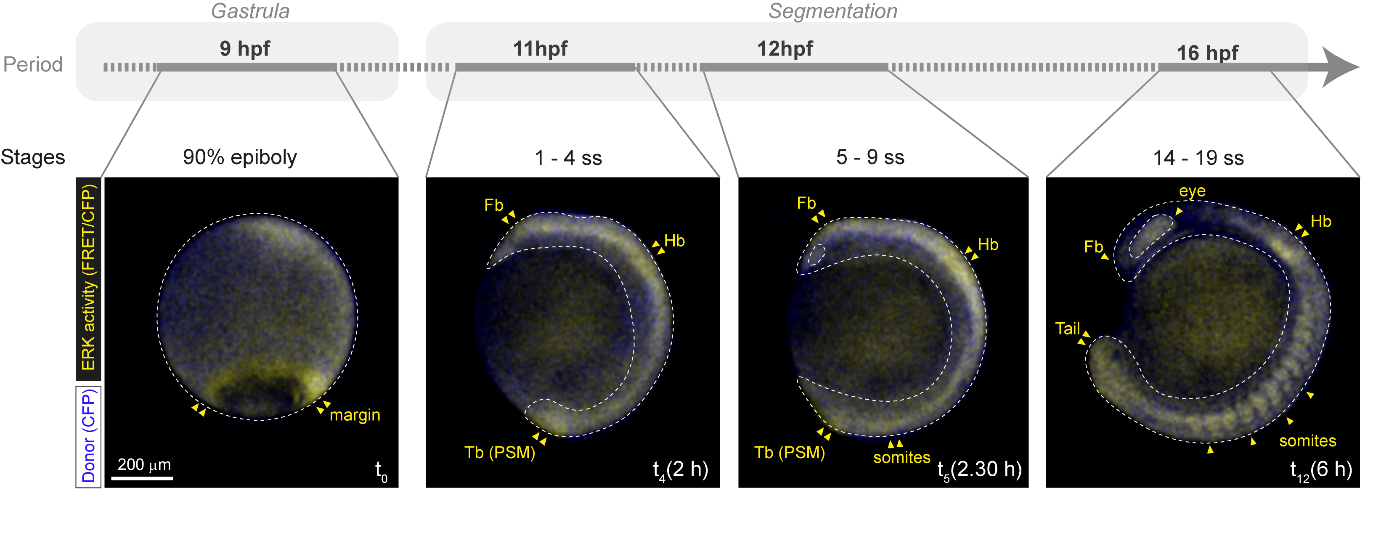


**Supplementary Figure 1. *In vivo* time lapse in *Teen* embryos obtained by spectral unmixing-FRET protocol reporting high ERK activity in zebrafish embryos from late gastrulation till segmentation stage.** Schematics depicting zebrafish early developmental stages from late gastrulation to somitogenesis in which pERK signal increase (FRET/CFP ratiometric image in yellow, indicated by arrows) was recorded using spectral unmixing FRET protocol in *Teen* embryos. Regions with high ERK activity are initially limited to gastrula margin (here at ~ 9 hpf), are observed in the tailbud/presomitic mesoderm (Tb PSM), forebrain (Fb) and hindbrain (Hb) (here at ~ 11 hpf) as well as in early born (here at ~ 12 hpf) and mature somites (here at~ 16 hpf). A dashed white line outlines the developing embryo. Donor (CFP) is shown in blue.


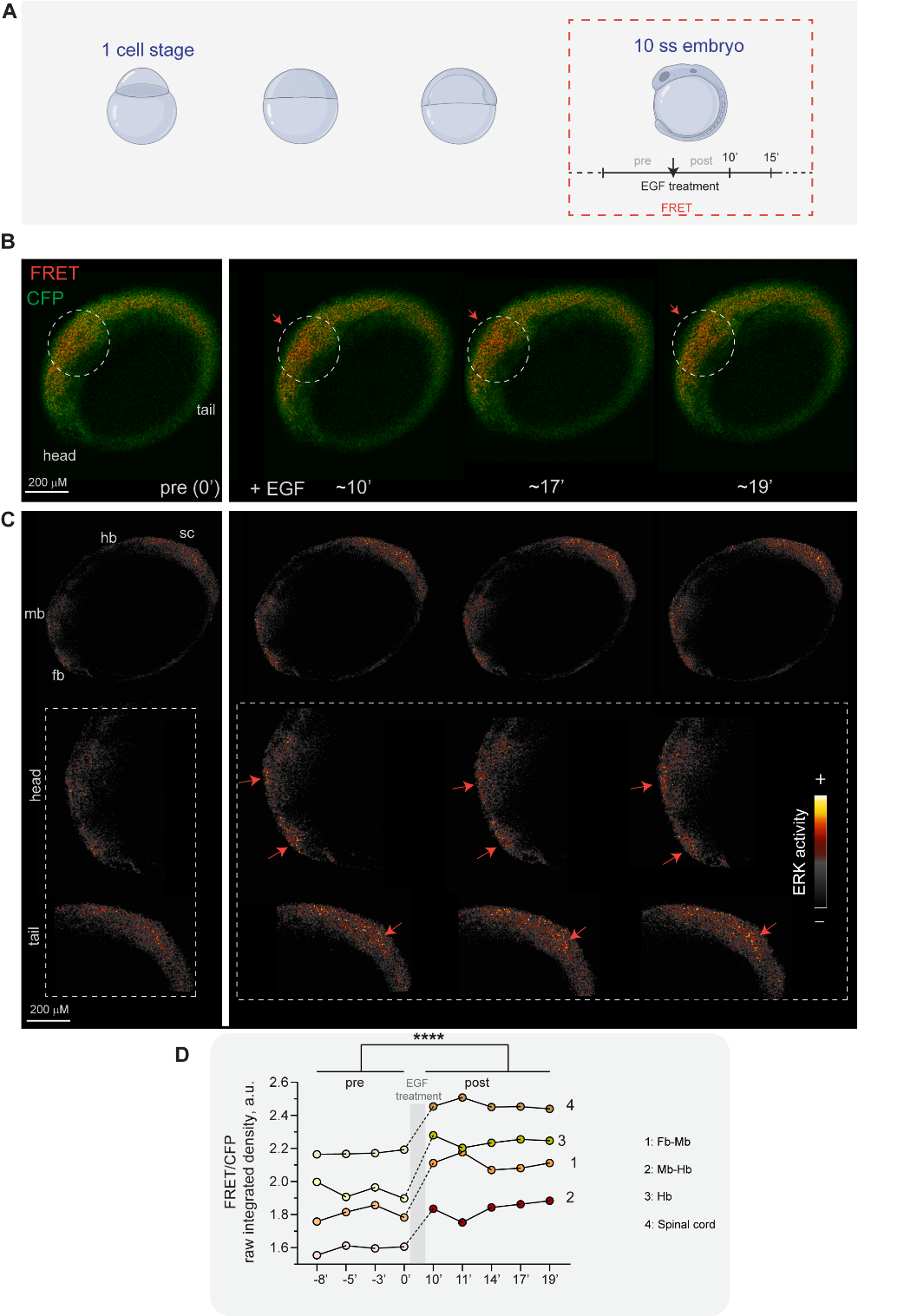


**Supplementary Figure 2. Live reporting of increased ERK activity captured in a zebrafish embryo at segmentation stage expressing the FRET-based ERK sensor *Teen* upon EGF stimulation.** (**A**) Schematics depicting zebrafish iearly development and circa 10 ss stage (red square) *Teen* embryos exposed to acute (10’) treatment with rat EGF and imaged by standard FRET imaging using donor excitation and collection of donor and acceptor emission spectra for a defined nm range (ratiometric module). (**B**) Sum-intensity projections of confocal x,y,z live scans of a single embryo, acquired with a time interval of ~1 min, showing FRET (red) and CFP (donor, green) signal before (pre, 0’) and at three different time points after EGF treatment ( 10’, 17’ and 19’) . Representative pERK signal increase (FRET channel) in the hindbrain region well visible by the embryo orientation is indicated by a dashed white circle. (**C**) Single superficial z-layer for the FRET channel at different time points rendered with the “smart” LUT intensity scale in Fiji. Fb, Mb, Hb: forebrain, midbrain, hindbrain, respectively. The middle and lower panels show magnification on “head” and “tail” regions, respectively, marked by a white dashed line. Areas of clear signal increase are indicated by red arrows. (**D**) The graph shows the quantification and statistical analysis of the fluorescence intensity (a.u. = arbitrary units) expressed as FRET/CFP at different time points in different regions of the developing nervous system. One-way ANOVA is used to assess statistical significance (**** p < 0.0001). Source data are provided as a Source Data file.


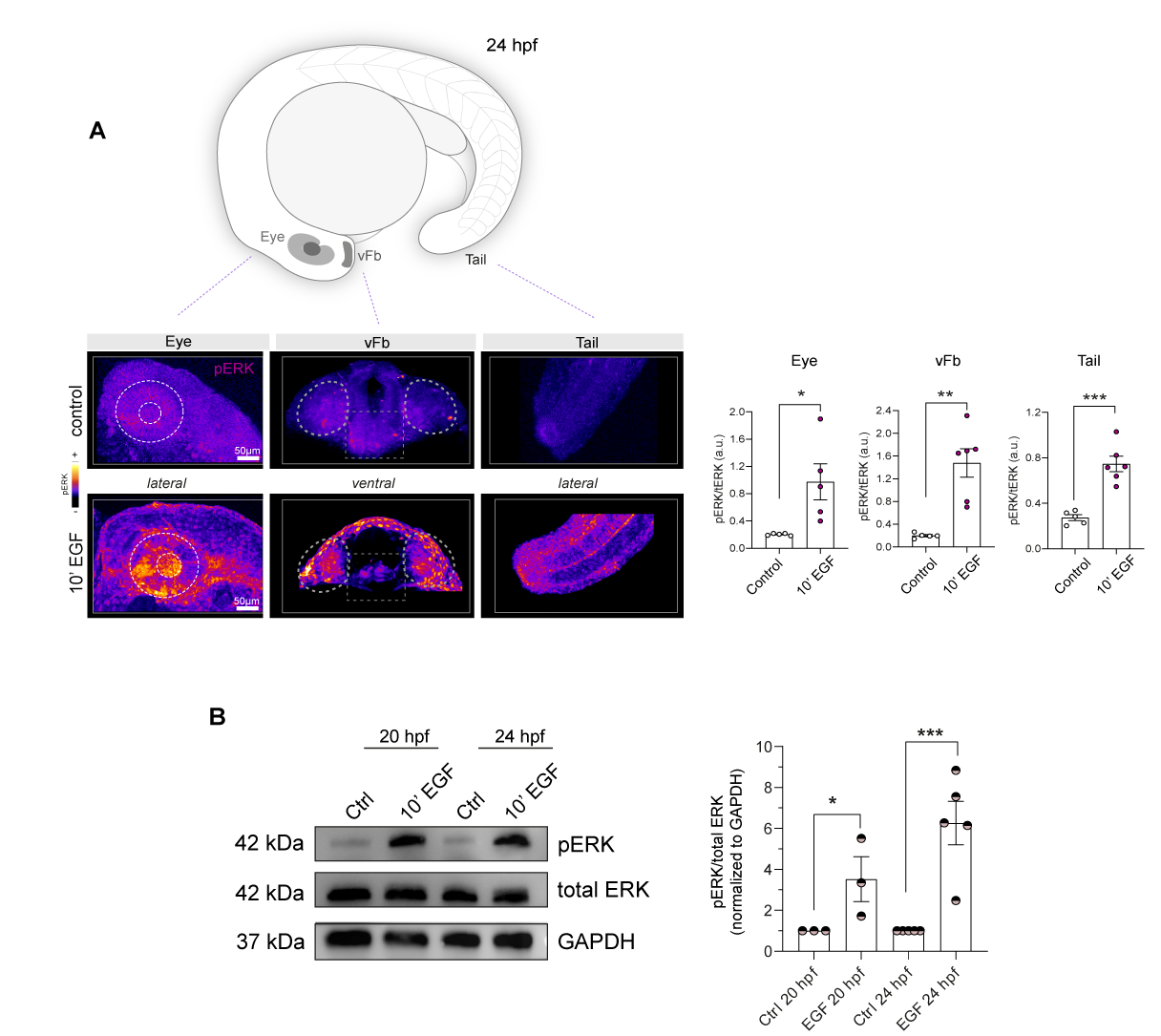


**Supplementary Figure 3**. **Increased pERK levels upon acute treatment of wild-type embryos with EGF is validated by immunofluorescence and western blot analysis.** (**A**) Immunofluorescence against phosphorylated ERK (pERK rendered in “fire” LUT) in 24 hpf treated with DMSO vehicle control (control) or with 10’ rat EGF by bath immersion. Increased signal in different regions is shown upon EGF treatment. VFb: ventral forebrain. Bar graphs showing quantification for specific regions

Bar graphs on the right show normalized pERK levels (pERK/tERK ratio, raw integrated density, a.u. = arbitrary units), calculated in the regions indicated (eyes, vFb, tail). Data are expressed as mean ± SEM. Ctrl: control (untreated embryos). For Ctrl N = 5; For EGF-treated embryos N = 5 (eye region) and 6 (vFb and tail regions). (**B**) Western blot detection of pERK from whole embryo extracts derived from 20hpf and 24 hpf embryos (untreated and upon 10’ EGF bath-exposure). The bar graph shows the densitometric analysis of the pERK band. pERK signal is normalized by the intensity of tERK relative to the levels of the loading control marker GAPDH. Data from three (20 hpf) and five (24 hpf) experiments are expressed as relative fold change to the loading control (mean ± SEM). In A and B, one-tailed Student's t test is used to assess the statistical significance (* p < 0.05, ** p < 0.01, *** p < 0.001) after outliers’ removal (ROUT method Q = 1%). Source data are provided as a Source Data file.

**
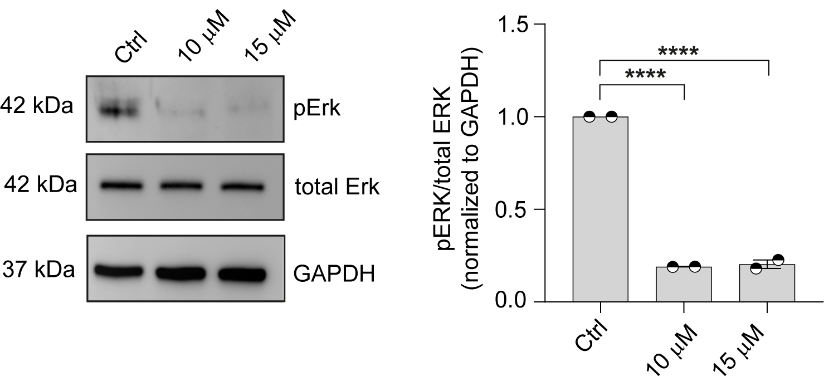
**

**Supplementary Figure 4.** **Decreased pERK signal upon chronic exposure with SHP099 in zebrafish embryos is validated by western blot analysis.** Western blot detection of pERK from 24 hpf whole embryo extracts after chronic treatment with vehicle control or SHP099 (between 4hpf and 24hpf) at two different concentrations. The bar graph shows the densitometric analysis of the pERK band. pERK signal is normalized by the intensity of total ERK relative to the levels of the loading control marker GAPDH. Reference molecular weights (kDa) are reported. Data from two replicates are expressed as relative fold change to the control (mean ± SEM). One-tailed Student's t test is used to assess the statistical significance (**** p < 0.0001). Source data are provided as a Source Data file.


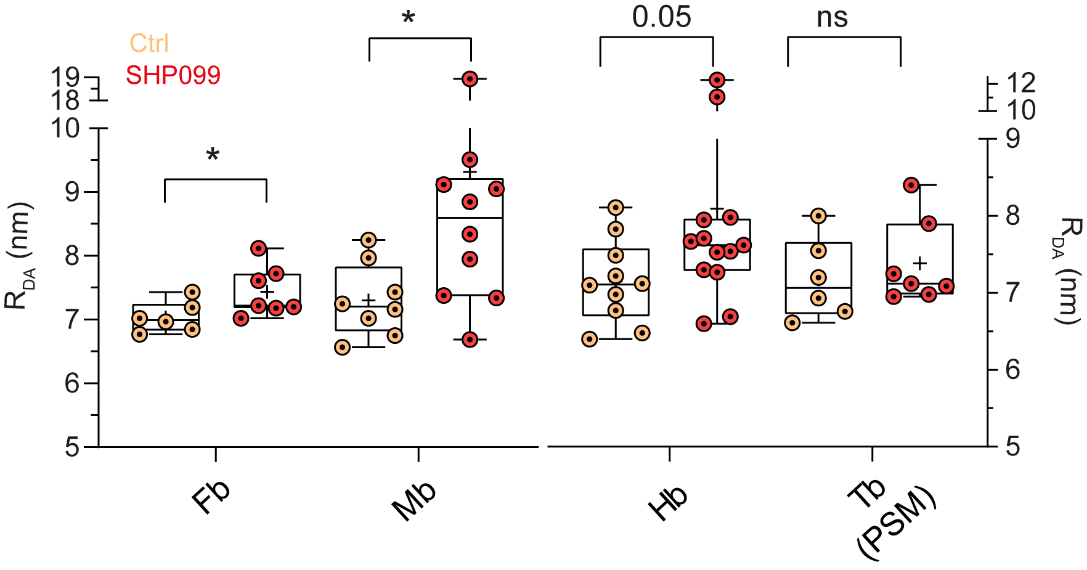


**Supplementary Figure 5. Increased R_DA_ values calculated from AB-FRET data of *Teen* embryos treated with SHP099.** R_DA_ values calculated from efficiency (E%) data of 24 hpf zebrafish expressing the FRET-based ERK sensor *Teen* upon prolonged SHP099 treatment. The box plot with median (middle line), 25th–75th percentiles (box), and min–max values (whiskers) shows the R_DA_ calculation (nm) in different regions. Fb: forebrain (n = 6 and 7 for control and treated), Mb: midbrain (n = 8 and 10 for control and treated), Hb: hindbrain (n = 10 and 13 for control and treated), Tb presomitic mesoderm (PSM), (n = 6 and 7 for control and treated). One-tailed Student’s t test is used to assess statistical significance (ns = not statistically significant, * p < 0.05). Source data are provided as a Source Data file.


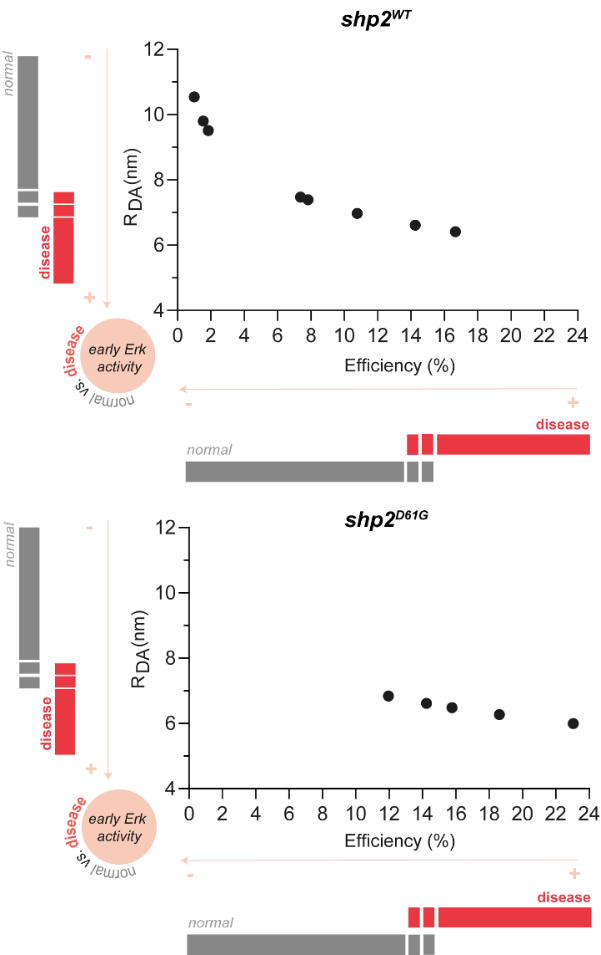


**Supplementary Figure 6. Visual correlation between AB-FRET efficiency (E %) and derived R_DA_ values in 5 hpf zebrafish *Teen* embryos over-expressing WT and mutant (D61G) Shp2*.*** The graphs summarize the R_DA_ (nm, y axis) relative to the efficiency (E %, x axis) quantified at the margin of 5hpf embryos for both Shp2^WT^ and Shp2^D61G^ groups (upper and lower graphs, respectively), correlating with ERK activity (data from Figure 4). The inversely proportional relationship between AB-FRET R_DA_ and efficiency values in both Shp2^WT^ and Shp2^D61G^ groups distinguishes normal (embryos overexpressing Shp2^WT^*,* upper graph) and disease (embryos overexpressing Shp2^D61G^, lower graph) conditions. Source data are provided as a Source Data file (Figure E’, E’’).


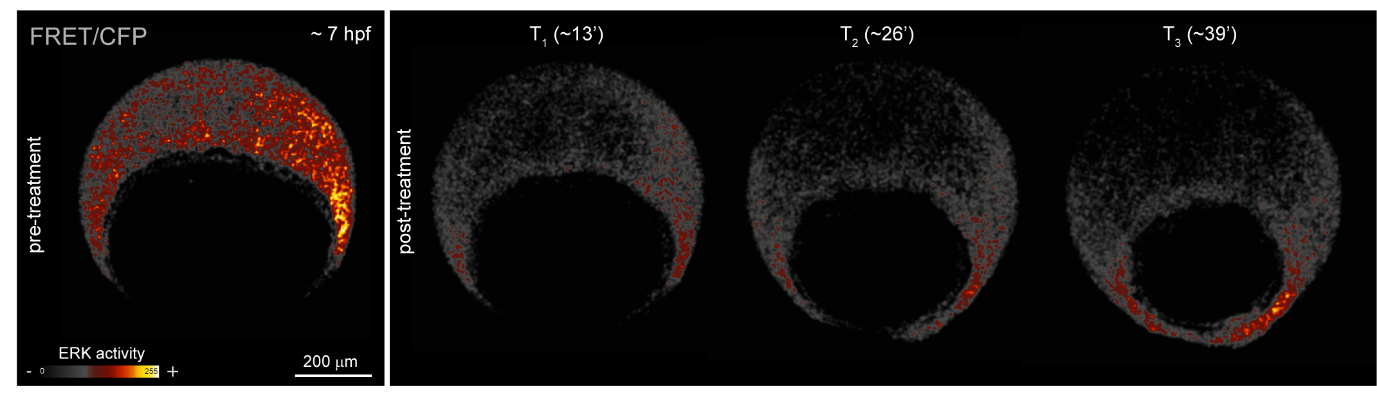


**Supplementary Figure 7.** **Live FRET of a control gastrula (7 hpf) before and after treatment with high-dose PD** . Maximum-intensity Z-stack projections showing consecutive images from a x,y,z,λ,t scan of a late gastrula (7hpf) before (pre-) and after (post-) treatment with high-dose PD. Spectral images are acquired with a time interval of 13’. T1: 13’, T2: 26’ and T3:39’ starting from the treatment with PD at 1 μM.


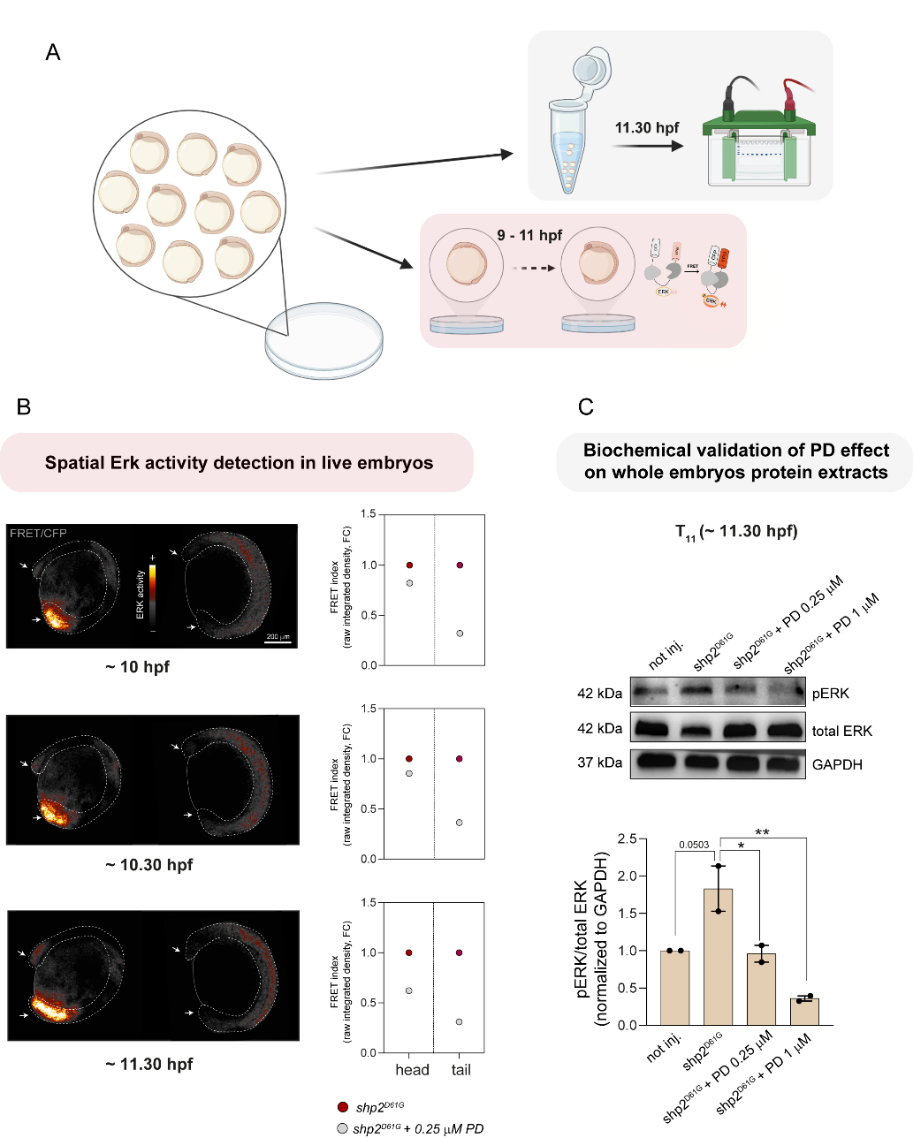


**Supplementary Figure 8. Decreased ERK signal in NS embryos (expressing Shp2^D61G^) treated with low-dose MEK inhibitor is measured by spectral unmixing-FRET during segmentation stages.** (**A**) Schematics depicting the workflow followed to assess ERK activity in live *Teen* embryos by spectral unmixing-FRET protocol (red square) and validated biochemically by western blot (gray square). (**B**) On the left, live sum-intensity projections of *Teen* embryos (expressing Shp2^D61G^ with or without treatment with low-dose PD0325901) between ~10 and ~11.30 hpf and imaged using spectral unmixing. Dashed white lines outline the embryos, white arrows indicate head and tail regions. On the right, quantification of the ERK signal change for FRET/CFP ratio of head and tail (raw integrated density, arbitrary units, a.u. is defined as “FRET index” and expressed as fold change, FC, of Shp2^D61G^ treated with low-dose PD, grey, *vs.* Shp2^D61G^, dark red). The ratiometric images (FRET/CFP) are rendered with the “smart” LUT intensity scale. (**C**) Representative blots and relative quantification of the pERK from whole embryo extracts derived from not injected, Shp2^D61G^ expressing embryos (~ 11.30 hpf) upon low-dose (0.25 µM) and high-dose (1 µM) PD treatment. The bar graph shows the densitometric analysis of the pERK band. pERK signal is normalized by the intensity of total ERK relative to the levels of the loading control marker GAPDH. Data from two independent experiments are expressed as relative fold change to the control (mean ± SEM). One-tailed Student's t test is used to assess the statistical significance (* p < 0.05, ** p < 0.01). Source data are provided as a Source Data file


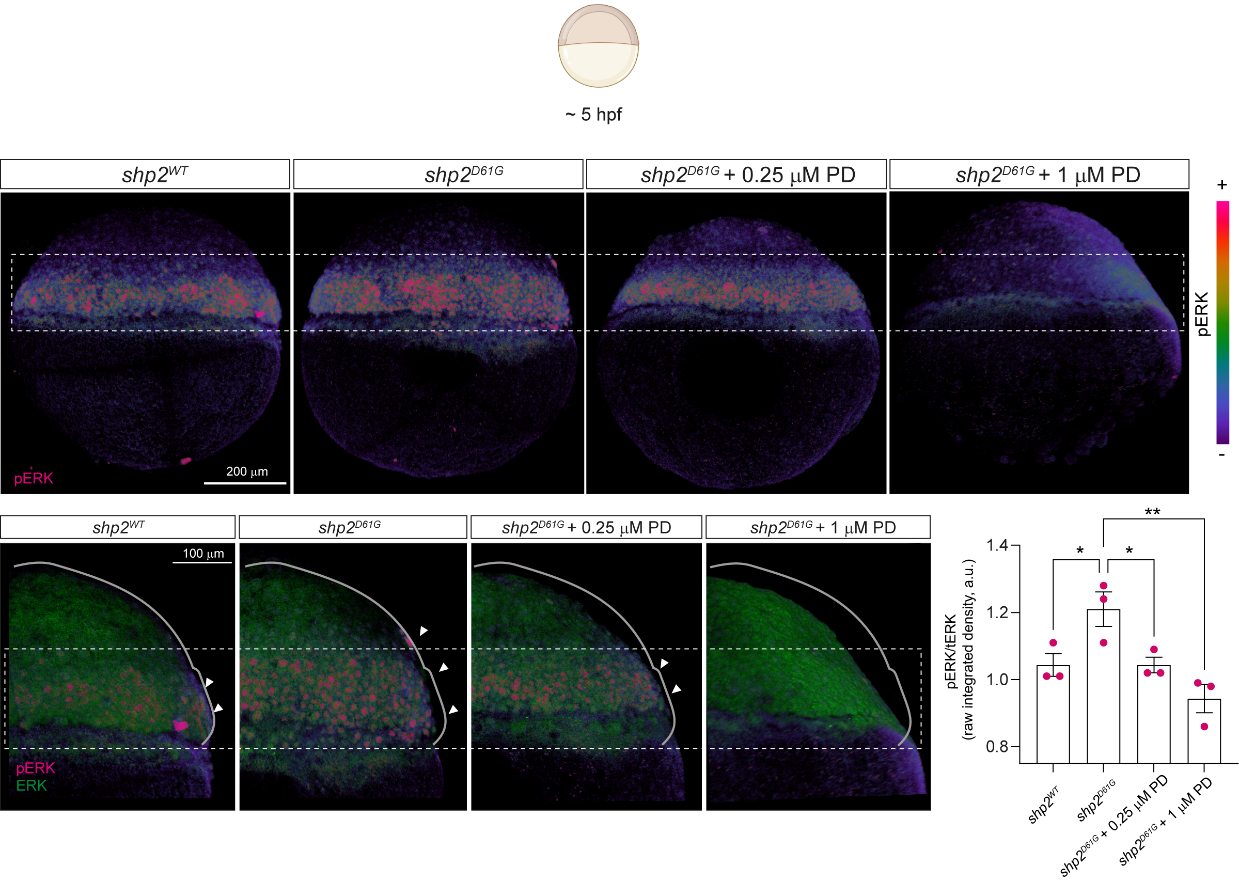


**Supplementary Figure 9.** **Immunofluorescence validation of the effect of low- and high-dose PD treatment in early NS fish models.** Confocal maximum intensity projections showing immunofluorescence results for pERK and relative quantification (normalized fluorescent intensity: pERK/tERK, phosphorylated/total ERK) in early gastrulae (5hpf) expressing WT and mutant (D61G) forms of Shp2 upon short treatment (from 4 to 5 hpf) of low-dose (0.25 µM) and high-dose (1 µM) PD. pERK is shown using “P.color #4” LUT (Leica palette) and magenta in the upper and lower panel, respectively, tERK is shown in green (lower panel). Increased pERK at the margin of the animal pole (white arrowheads) is rescued by PD treatment in a dose-dependent manner. Dashed white line indicates the margin region selected for quantification. White line depicts animal pole boundary. Bar graphs on the right showing normalized pERK levels (pERK/tERK ratio, raw integrated density, a.u. = arbitrary units), calculated in the margin zone of the animal pole. Data are expressed as mean ± SEM; n of embryos = 3. One-way ANOVA with Dunnett’s *post hoc* test is used to assess the statistical significance (* p < 0.05, ** p < 0.01). Source data are provided as a Source Data file.
